# High fat diet is associated with gut microbiota dysbiosis and decreased gut microbial derived metabolites related to metabolic health in young Göttingen Minipigs

**DOI:** 10.1371/journal.pone.0298602

**Published:** 2024-03-01

**Authors:** Ditte Olsen Lützhøft, Cecilie Bækgård, Elizabeth Wimborne, Ellen Marie Straarup, Karen-Margrethe Pedersen, Jonathan R. Swann, Henrik Duelund Pedersen, Kim Kristensen, Line Morgills, Dennis Sandris Nielsen, Axel Kornerup Hansen, Marianne Kronborg Bracken, Susanna Cirera, Berit Østergaard Christoffersen

**Affiliations:** 1 Department of Veterinary and Animal Sciences, Faculty of Health and Medical Sciences, University of Copenhagen, Frederiksberg C, Denmark; 2 School of Human Development and Health, Faculty of Medicine, University of Southampton, Southampton, United Kingdom; 3 Novo Nordisk A/S, Maaloev, Denmark; 4 Ellegaard Göttingen Minipigs A/S, Dalmose, Denmark; 5 Department of Food Science, Faculty of Science, University of Copenhagen, Frederiksberg C, Denmark; 6 Scantox A/S, Lille Skensved, Denmark; University of Louisville, UNITED STATES

## Abstract

The objectives were 1) to characterize a Göttingen Minipig model of metabolic syndrome regarding its colon microbiota and circulating microbial products, and 2) to assess whether ovariectomized female and castrated male minipigs show similar phenotypes. Twenty-four nine-week-old Göttingen Minipigs were allocated to four groups based on sex and diet: ovariectomized females and castrated males fed either chow or high-fat diet (HFD) for 12 weeks. At study end, body composition and plasma biomarkers were measured, and a mixed meal tolerance test (MMT) and an intravenous glucose tolerance test (IVGTT) were performed. The HFD groups had significantly higher weight gain, fat percentage, fasting plasma insulin and glucagon compared to the chow groups. Homeostatic model assessment of insulin resistance index (HOMA-IR) was increased and glucose effectiveness derived from the IVGTT and Matsuda´s insulin sensitivity index from the MMT were decreased in the HFD groups. The HFD groups displayed dyslipidemia, with significantly increased total-, LDL- and HDL-cholesterol, and decreased HDL/non-HDL cholesterol ratio. The colon microbiota of HFD minipigs clearly differed from the lean controls (GuniFrac distance matrix). The main bacteria families driving this separation were *Clostridiaceae*, *Fibrobacteraceae*, *Flavobacteriaceae* and *Porphyromonadaceae*. Moreover, the species richness was significantly decreased by HFD. In addition, HFD decreased the circulating level of short chain fatty acids and beneficial microbial metabolites hippuric acid, xanthine and trigonelline, while increasing the level of branched chain amino acids. Six and nine metabolically relevant genes were differentially expressed between chow-fed and HFD-fed animals in liver and omental adipose tissue, respectively. The HFD-fed pigs presented with metabolic syndrome, gut microbial dysbiosis and a marked decrease in healthy gut microbial products and thus displayed marked parallels to human obesity and insulin resistance. HFD-fed Göttingen Minipig therefore represents a relevant animal model for studying host-microbiota interactions. No significant differences between the castrated and ovariectomized minipigs were observed.

## Introduction

Obesity is one of the most frequent non-communicable diseases and the prevalence has tripled since 1975 [1]. In 2016, over 1.9 billion adults worldwide were overweight and of those 650 million were obese [1]. A frequent consequence of obesity is metabolic syndrome, which is characterized by dyslipidemia with elevated triglycerides, total cholesterol, low density lipoprotein cholesterol (LDL-C), and decreased high-density lipoprotein cholesterol (HDL-C), in addition to insulin resistance [2, 3].

In the development of obesity gut microbiota dysbiosis is a contributing factor [4], which influences metabolic health, host energy metabolism and immune functions [5]. A gut microbiota characterized by low degree of richness and dominance of pathobiont bacteria including *Enterobacteriaceae* and *Erysipelotrichaceae* is associated with an obese phenotype [6]. In addition, decreased intake and/or decreased microbial fermentation of indigestible complex carbohydrates in insulin resistant and obese patients may result in a lower production of short chain fatty acids (SCFA) [7]. In the healthy gut, microbiota-derived SCFAs exert multiple beneficial functions, spanning from energy supply for colonocytes to regulation of appetite, lipid synthesis, and glucose metabolism through free fatty acid receptors 2 and 3 and AMP-activated protein kinase signaling [8], all of which are altered in obesity. In addition, the obesity associated gut microbiota may produce increased amounts of branched chain amino acids (BCAA), and these have been associated with the development of obesity and type 2 diabetes mellitus [9, 10]. Increased gut microbiota-derived BCAA synthesis combined with reduced microbial BCAA uptake and catabolism in the obese insulin resistant state results in accumulation of these amino acids in circulation [9, 11]. This may potentially cause persistent activation of the mammalian target of rapamycin complex 1 (mTORC1), which leads to decreased insulin signaling and contributes to the development of insulin resistance [9]. Furthermore, BCAAs are implicated in lipid and glucose metabolism, and immune functions [12]. In fact, several studies have suggested that both inadequate and excessive levels of BCAA have negative consequences for obesity and its comorbidities [12].

Understanding the complex interplay between the host and the gut microbiota and its products can increase the understanding of the pathogenesis of obesity, insulin resistance and potentially point towards new treatment modalities for obesity and its complications.

To investigate the molecular mechanisms and other aspects of obesity, animal models are necessary, and the Göttingen Minipig is useful as non-rodent obesity model due to its many physiological and anatomical similarities with humans, the small size and the high degree of standardization and characterization [13]. The model has already been used to study the effects of obesity, metabolic syndrome, nonalcoholic steatohepatitis, diabetes [14–16], and chronic inflammation associated with obesity [17]. A few studies have focused on gut microbiota in various minipig models. These studies support the minipig as a relevant model to study host-microbiota interaction [18–20].

The primary aim of this study was to characterize a diet-induced obese adolescent Göttingen Minipig model for testing new pharmaceutical anti-obesity treatments and for studying the physiological and metabolic changes occurring with obesity. In this study, obesity was induced by feeding an excess of a diet high in fat and fructose, while the control groups were fed a normal low fat chow diet restrictedly in order to mimic an unhealthy vs. a healthy diet. Specifically, changes in the gut microbiota and gut microbial products and their relation to the metabolic phenotype were studied. A secondary objective was to compare the metabolic phenotype of ovariectomized female and castrated male minipigs.

## Material and methods

### Ethics

The animal study was carried out in accordance with the Danish Animal Experimentation Act (LBK 1107 from 02/07/2022) and the EU directive 2010/63/EU, and was approved by the Animal Experiments Inspectorate, Ministry of Food, Fisheries and Agriculture, Denmark.

All surgeries were performed under appropriate anesthesia and analgesia followed by appropriate postoperative treatment as described in S1 File in order to minimize pain and suffering.

### Animal study

**Fig 1:**
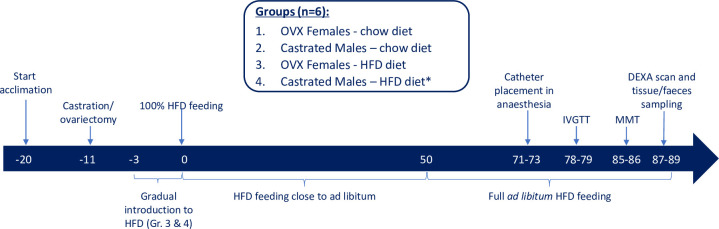
Study and group overview. DEXA: dual-energy X-ray absorptiometry, HFD: High fat diet, OVX: ovariectomised, IVGTT: Intravenous glucose tolerance test, MMT: Mixed meal test. Days on x-axis. * One male minipig on HFD died prematurely, leaving 5 minipigs in this group. For practical reasons, the blood samples, metabolic tests and tissue sampling were done over several days, but with animals from each group represented on each test day in order to avoid day-to-day bias.

The overall study design is shown in Fig 1. Twelve young male and 12 young female Göttingen Minipigs (Ellegaard Göttingen Minipigs A/S, Dalmose, Denmark) were either castrated or ovariectomized (OVX) 11 days prior to study start at which point they were 9 weeks of age. Neutering was performed since it has previously been described that both OVX female Göttingen Minipigs and castrated male Göttingen Minipigs develop severe obesity when fed either a high energy or a chow diet *ad libitum*, whereas intact males do not develop severe obesity [16, 21, 22]. See S1 Table for diets composition and S1 File for further details regarding the surgical procedure.

Three days prior to study, the pigs were allocated to groups based on body weight (BW) so that the groups had a similar average BW at start of diet feeding. Six female and 6 male minipigs were gradually shifted from chow diet (SDS Minipig expanded, Special Diets Services, Scanbur, DK) to high fat diet (HFD, custom-made at Foulum, Aarhus University, Denmark) and continued on this diet for the full 12 weeks duration of the study. The HFD diet was fed close to *ad libitum* for the first seven weeks to avoid overeating and too much food spillage during group housing, and *ad libitum* for the last five weeks of the study. The remaining 6 female and 6 male minipigs were fed restrictedly on the standard minipig chow diet twice daily according to the breeder’s recommendation in order to follow the normal growth curve of Göttingen Minipigs (www.minipigs.dk). The daily food intake in these animals ranged from 220–250 g/day during the study. The HFD contained 25% fat (primarily lard) and with 23% fructose and 0.5% cholesterol added. The two diets were not matched with respect to the individual components other than the protein content, which was 13% in both diets, since the purpose of this study was to investigate the effects of consuming a calorie-dense Western type of diet with low fiber content and high amounts of saturated fat and refined sugar, rather than to directly investigate the effect of changing specific diet components. For detailed diet specifications, see S1 Table.

The minipigs were group housed in the first seven weeks and single housed in the last five weeks due to measurement of individual food intake and the presence of central-venous catheters. The room temperature was 21°C ± 3°C, with cycles of 12 hours of light followed by 12 hours of darkness. All animals had free access to tap water. At end of the study the animals were anesthetized in the fasted state with 1 mL/10-15 kg i.m. injection of Zoletil mixture (S1 File) before being euthanized by exsanguination.

### Body weight and body composition

Body weight (BW) was obtained once a week throughout the study using a floor scale. Body composition was determined at the end of the study in 18 hours fasted anaesthetized animals using dual-energy x-ray absorptiometry scanning (DEXA-scanning) (Hologic Explorer, Santax Medico, Aarhus, Denmark).

### Intravenous glucose tolerance test (IVGTT)

Ten days before the end of the study an IVGTT was performed in 18 hours fasted animals. A glucose bolus of 0.3 g/kg (0.6 mL/kg, 500 g/L, Glucose SAD) was given as an intravenous bolus through a central venous catheter, which was placed via an ear vein under general anaesthesia (S1 File). Blood samples were collected at the following time points in relation to the glucose bolus: Predose, 2, 5, 7, 15, 20, 25, 39, 40, 50, 60, 75, 90, 105 and 120 minutes post dose. The samples were stored on wet ice for a maximum of 30 minutes until centrifugation at 2000×g for 10 min at 4°C. The resulting plasma was stored at either -20°C or -80°C until analysis.

### Mixed meal test (MMT)

A MMT was performed approximately 4 days prior to euthanasia to evaluate oral glucose tolerance. The pigs were fed 6 g of the mixed meal per kg BW. This amount of diet gives rise to the following amounts of macronutrient intake per kg BW: 0.24 g protein/kg BW, 0.76 g fat/kg BW and 2.1 g sucrose/kg BW. The remaining 2.9 g of the 6 g mixed meal given per kg BW was water. The minipigs were given 20 min to finish the meal after which the timer was started. Blood (2 mL) was sampled at the following time points in relation to the end of the meal: 0, 15, 30, 45, 60, 90, 120, 150, and 180 minutes. The blood samples were transferred to EDTA tubes with 25 μl special stabilization buffer/ml blood (S1 File). The blood samples were stored on wet ice until centrifugation at approximately 2000×g for 10 min at 4°C within max. 30 min after sampling. The resulting plasma was kept at either -20°C or -80°C until analysis was performed.

### Fecal and tissue samples

Fecal samples from the colon were collected upon euthanasia from the colon spiral junction and placed at -80°C. Collection tools were sterilized between each animal using 70% ethanol. For gene expression analysis, liver, omental adipose tissue, and skeletal muscle tissues were collected at euthanasia and immediately frozen at -80°C.

For histopathological examination the following tissues were collected and fixed in phosphate-buffered neutral 4% formaldehyde: Liver, lung, kidneys, heart, pancreas, and white adipose tissue (visceral/omental and subcutaneous inguinal). The tissues were trimmed, embedded in paraffin, cut at a nominal thickness of 5 μm, stained with haematoxylin and eosin, and examined under a light microscope.

### RNA isolation and gene expression analysis

RNA was extracted from ~30 mg of liver using the RNeasy Mini Kit (Qiagen), from 100 mg of omental fat tissue using the protocol of Skallerup et al. [23] and from 100 mg skeletal muscle tissue using the RNeasy Fibrous Tissue Mini Kit (Qiagen) (details in S1 File).

A panel of 96 different obesity relevant genes including possible reference genes were chosen from an *in-house* primer library (S2 Table) for profiling by qPCR using the high-throughput qPCR Biomark HD platform (Fluidigm, San Francisco, California) following manufacturer’s protocols. Additionally, 7 extra genes (S2 Table, genes marked with a *) of interest were profiled solely in liver samples using QuantiFast SYBR® Green PCR Kit (Qiagen, Germany) in a Mx3005P platform as previously described [24].

### Total DNA extraction, sequencing, and pre-processing of raw data

Extraction of total DNA from fecal samples was performed by using Bead-beat micro-AX bacteria gravity kit (A&A biotechnology, Gdynia, Poland) according to manufacturer’s instruction. The bacterial composition of the colon microbiota was determined using Illumina Next generation (NextSeq) high throughput sequencing of the 16S rRNA gene V3-region as described in detail by Krych and colleagues [25]. In brief, amplified fragments containing adaptors and tags were purified and the following normalization was performed using custom made beads. Hereafter, samples were pooled and subjected to 150 bp paired-end Illumina NextSeq sequencing of the V3 region of the 16S rRNA gene. The raw dataset containing pair-ended reads was merged and trimmed, with a subsequent de-replication, purging of chimeric reads and high quality (97% similarity level) operational taxonomic units (OTUs) were constructed. Taxonomically assignments were performed using sintax coupled to the EZtaxon 16S rRNA gene reference database. The raw reads were filtered and low-abundance OTUs across all samples with below 0.005% in abundance were removed using R software V 4.1.1. The sequencing depth was on average 71,772 reads per sample before filtering and 66,665 after filtering. The differences in sequencing depth were corrected using Cumulative Sum Scaling normalization *via* MetagenomeSeq 1.32.0 [26].

### Clinical chemistry and serum/plasma analyses

Standard clinical chemistry parameters (measured in serum), fructosamine, haptoglobin, total bile acids (TBA) (measured in EDTA plasma), free fatty acids (FFA), and glycerol (measured in EDTA+NaF plasma) were analyzed on a Cobas 6000® autoanalyzer (Roche Diagnostics GmbH, Mannheim, Germany) according to the manufacturer´s instructions. Plasma glucose was analyzed on a Biosen S-Line Lab autoanalyzer according to the manufacturer´s instruction (EKF Diagnostics, Ebendorfer Chaussee 3, 39179 Barleben, Germany). Insulin and glucagon concentrations were determined by Luminescent Oxygen Channeling Immunoassay as described by Pedersen et al. [27].

Imidazole propionate was quantified using ultra-performance liquid chromatography coupled to tandem mass spectrometry according to previous work. Briefly, plasma samples were extracted with 3 volumes of ice-cold acetonitrile containing internal standards (13C3-labeled ImP and urocanate). After derivatization to butyl esters using 5% hydrochloric acid in butanol, the samples were separated on a C18 column using a gradient consisting of water and acetonitrile. Quantification was made using an external calibration curve [28].

SCFA were analysed as follows: Sample derivatization: 10 μl sample, 10 μl methanol (75%), 10 μl 200 mM 3-NPH (3-nitrophenylhydrazine in MeOH (75%)), 10 μl 120 mM EDC-6% pyridine (N-(3-Dimethylaminopropyl)-N-ethylcarbidiimide in 75% MeOH with 6% pyridine) was mixed and incubated 45 min at room temperature (RT) with shaking. The derivatization reaction was quenched with 10 μl of 200 mM quinic acid in MeOH, mixed and incubated 15 min, RT with shaking. 950 μl of water was added to the samples followed by centrifugation at 15,000×g, RT, for 5 min. 100 μl of supernatant and 100 μl of internal standard (13C 3-nitrophenylhydrazine labeled short chain fatty acids) transferred to HPLC vials. Samples were analyzed immediately or kept at 4°C until analysis.

The samples were analyzed with LC-MS/MS consisting of an ExionLC UHPLC system coupled to a 6500+ QTRAP (both from AB Sciex LLC, Framingham, USA). The analytes were separated in a Phenomenex Kinetex C18 (100 × 2.1 mm, 1.7 μm, 100 Å) column, at 40°C, following gradient: 0–3 min 0.5% B, 3.00–3.01 min 0.5–2.5% B, 3.01–6.00 min 2.5–17% B, 6.00–10.00 min 17–45% B and 10.00–13.00 min 45–55% B, followed by washing and re-equilibration of the column. Mobile phase A and B were water and acetonitrile, respectively, and total flow was set to 0.4 mL/min. APCI ionization was used in positive polarity and the analytes were detected using optimized MRM-transitions for each analyte and internal standard. A calibration curve covering the range of the analytes in the samples was injected together with the analytes.

### Metabolomic profiling

A range of low-molecular weight metabolites (126 features) and lipids (374 features) were measured in the plasma samples (6 HFD females, 6 chow-fed females, 5 HFD males, 3 chow-fed males; the samples from the three remaining chow-fed males were unfortunately lost due to a technical error during the analysis and one HFD male died prematurely before the blood sampling time point (see below)) using Biocrates MxP Quant 500 kits with a Waters Xevo TQ-XS equipped with an ACQUITY Premier UPLC system. The kits were prepared and analyzed following the manufacturer’s instructions. A range of metabolism indicators (232 sums and ratios of various metabolites) were calculated from the metabolomic data using the MetaboINDICATOR software tool from Biocrates. The metabolomic profiles and these indicators were imported separately into MetaboAnalyst (www.metaboanalyst.ca). Variables with greater than 80% missing values (*i*.*e*., those below the limit of quantification) across the sample set were excluded. For those metabolites with less than 80% missing values, the missing values were replaced with 1/5 of the minimum positive value for that metabolic feature across the sample set. All metabolites were log transformed before further data analysis. For the indicator dataset, any variables with a missing value were excluded.

### Calculations and statistics

For IVGTT data, insulin sensitivity index was calculated using a non-linear mixed effect approach for minimal model-derived insulin sensitivity index (SI) and glucose-mediated glucose clearance (SG) [29, 30]. Homeostatic model assessment of insulin resistance (HOMA-IR) was calculated based on fasting glucose and insulin values from the IVGTT according to the following formula: *HOMA-IR = fasting plasma glucose [mM] x fasting plasma insulin [μU/ml]/22*.*5* [31]. The Matsuda insulin sensitivity index [32] was calculated for the IVGTT using time 0, 30, 60 and 120 minutes.

Statistical analysis of physiological and biochemical measurements and data visualization was done in GraphPad Prism v. 9.0.1 (GraphPad Software, San Diego, California USA, www.graphpad.com) and GGplot2 v3.3.3 (Hadley Wickham, Auckland, New Zealand). The impact of the variables diet and sex was evaluated by two-way ANOVA followed by Tukey or Bonferroni correction for multiple testing, and *P*-values <0.05 were considered significant. Evaluation of data normality was performed using QQ plots and Shapiro-Wilks test, and homogeneity of variance was evaluated using Spearman´s test for heteroscedasticity (R´s of predicted value vs [residual]). Data were transformed as appropriate to obtain normally distributed residuals.

For conducting bioinformatic analysis the 16S rRNA gene amplicon sequencing data, R software v4.1.1 and packages Vegan v2.5.7, PhyloSeq v1.34.0, MetagenomeSeq v1.32.0, GUniFrac v1.1, and DAtest v2.7.17 were used. Differences in bacterial abundance between groups were detected using DAtest package (DESeq2) having diet as predictor variable and sex as co-variable. Moreover, bacterial differences between groups were also investigated using https://huttenhower.sph.harvard.edu/lefse/ for conducting a Linear Discriminant Analysis (LDA) Effect Size (LEfSe) using default settings [33] (apart from LDA threshold), which included setting the threshold on the logarithmic LDA score for discriminative features to 2.5 and Kruskal-Wallis test for classes reached significance when p < 0.05. The filtering of taxa was adjusted to exclude OTUs below 0.01% in relative abundance for the analysis of bacterial differences between groups. Differential clustering (beta-diversity based on GUniFrac-metrics) was evaluated using ANOSIM (PERMANOVA). Alpha-diversity using Chao1 index was evaluated using two-way ANOVA having diet and sex as variables.

For metabolomic profiling univariate statistics were used to identify pair-wise metabolic differences between the HFD and chow groups. This was achieved using a t-test with a false discovery rate (FDR)-adjusted *P*<0.05 threshold to account for multiple testing [34]. The data were visualized in a volcano plot and a two-fold change threshold was used to highlight biologically relevant differences.

Gene expression data were processed using Genex Pro v.6 software (MultiId, Sweden). Briefly, Cq values from both chips were calibrated for differences in baseline correction (only for the Biomark platform data), corrected for PCR efficiency and normalized to the reference genes selected for each tissue; subsequently, cDNA replicates were averaged, fold changes were calculated related to SD group and data was log2 transformed prior to statistical analysis. Firstly, a two-way ANOVA test was applied for each tissue profiled (with diet and sex as factors) followed by Bonferroni’s multiple test correction (MTC); but because there were no significant differences between sexes, a t-test was subsequently applied to compare chow versus HFD groups for each tissue (S2 Table, 4 sheets showing t-test results).

## Results

One HFD-fed male minipig unexpectedly died for unknown reasons, and without showing previous signs of disease, on study day 50. Data from this animal has been excluded. Due to catheter dysfunction in one male in the chow-fed group and one female and two males in the HFD-fed groups, it was not possible to obtain blood samples from all animals during the MMT.

### High fat diet increased body weight and fat storage and contributed to poorer glycemic control and dyslipidemia

**Fig 2:**
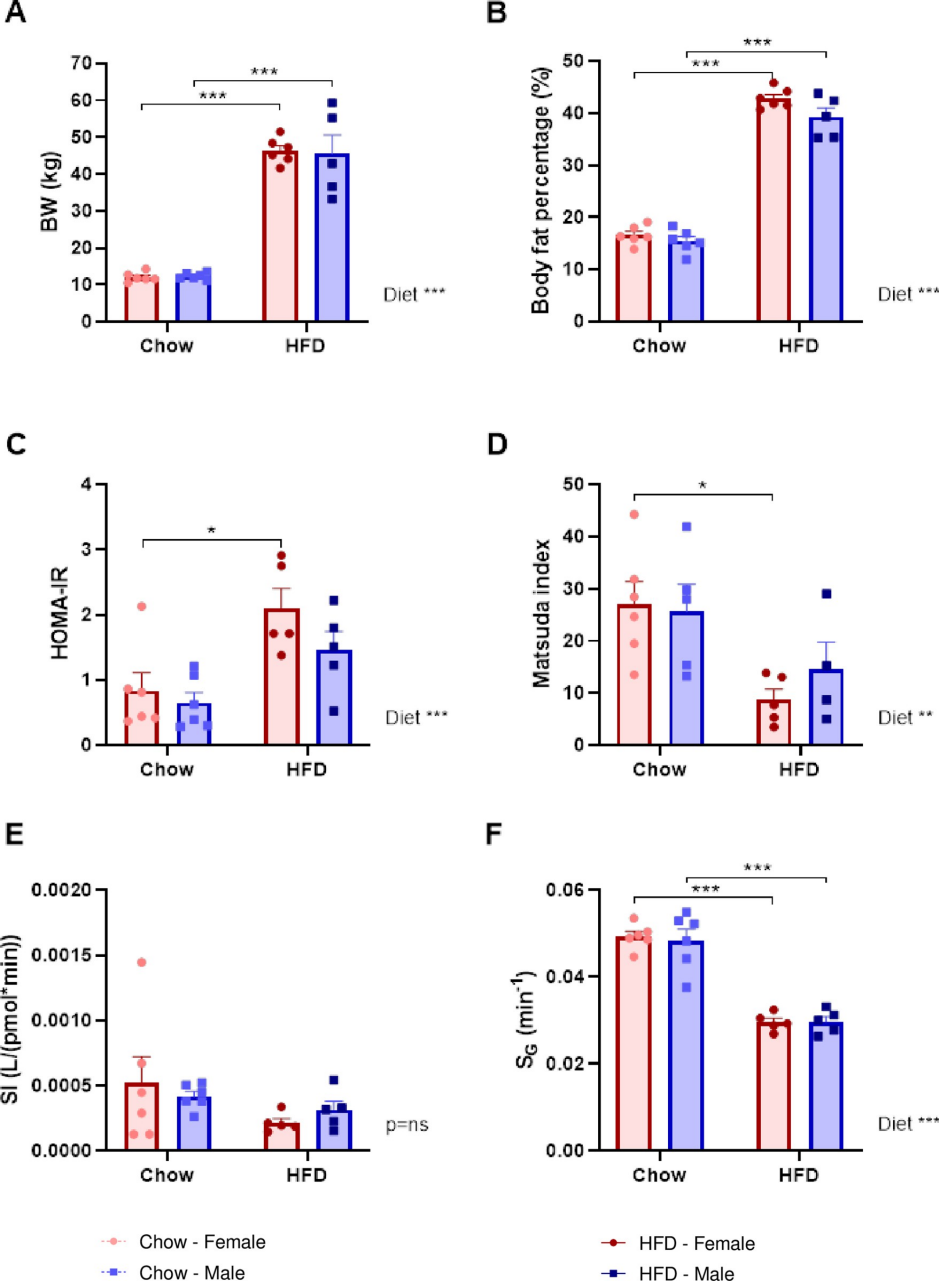
Body weight, body composition and glucose metabolism data. Twenty-three female and male Göttingen Minipigs were fed either chow (female n = 6, male n = 6) or HFD (female n = 6, male n = 5) for 12 weeks starting at 9 weeks of age. **A.** End study Body Weight (kg); **B.** Body fat (%); **C.** HOMA-IR, **D.** Matsuda´s insulin sensitivity index from the MMT; **E.** Insulin sensitivity **(**SI) and **F**. Glucose effectiveness (SG) from minimal modeling. Light red and red circles are females fed chow or HFD. Light blue and dark blue squares are males fed chow or HFD. Data are shown as bar plots with individual data points and mean±SEM. Significant effect of diet and sex was determined using two-way ANOVA followed by Tukey´s multiple comparison test. *P<0.05, **P<0.01 and ***P<0.001 mark the level of statistical significance.

After 13 weeks the HFD-fed minipigs had approx. 2.4 times higher BW than the chow-fed minipigs and a body fat percentage of around 40%, with no significant difference between the two sexes (Fig 2A and 2B). At the end of the study, both fasting glucose, insulin and glucagon levels were overall significantly elevated in the HFD pigs, whereas fasting fructosamine (a marker of medium-term glucose control in pigs [35]), did not differ between the groups (S3 Table).

Insulin sensitivity estimated by HOMA-IR and the Matsuda index during MMT revealed that HFD-feeding resulted in a significantly increased insulin resistance compared to chow (Fig 2C and 2D). This effect was only statistically significant in the female minipigs after correction for multiple comparisons. In contrast, the IVGTT-derived insulin sensitivity index, SI, was only slightly and non-significantly reduced in the HFD-fed minipigs (Fig 2E), whereas glucose effectiveness, SG, was significantly decreased in the HFD minipigs (Fig 2F). There were no significant differences in the MMT and IVGTT parameters between OVX females and castrated males.

Furthermore, the HFD increased circulating total cholesterol as well as HDL- and non-HDL-cholesterol. However, the non-HDL-cholesterol increased relatively more, leading to a significantly lower HDL-cholesterol/non-HDL-cholesterol ratio in the HFD fed animals (S3 Table).

### High fat diet induced a shift in the colon microbiota

**Fig 3:**
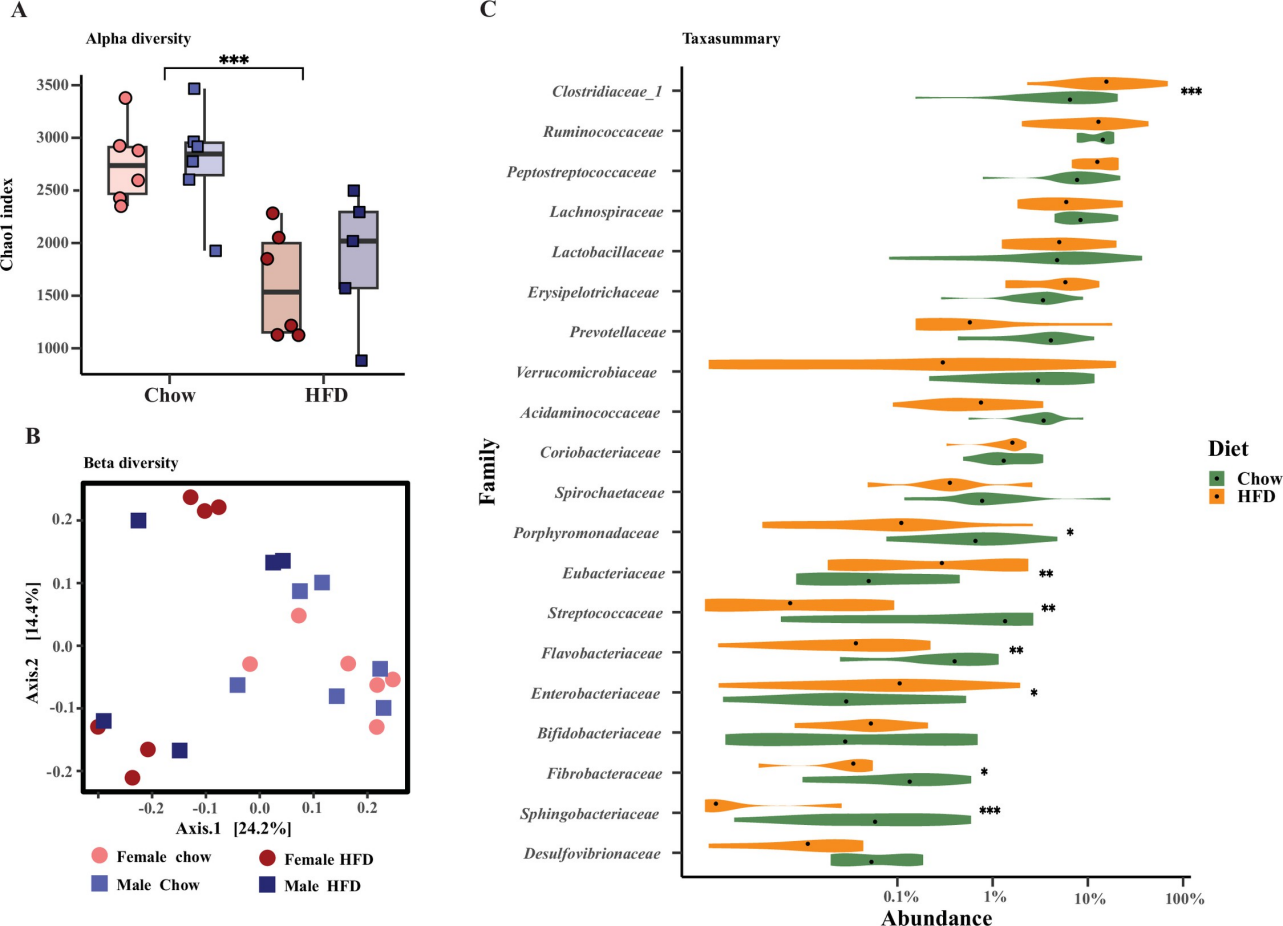
Colon microbiome. Colon microbiome from twenty-three female and male Göttingen Minipigs fed either chow or HFD (n = 5–6) for 12 weeks determined using 16S rRNA gene amplicon sequencing. **A**. Boxplot showing Alpha diversity (Chao1 index) (25th percentile, 75th percentile, median, whiskers indicate max and min value; two-way ANOVA: diet: *P* = 0.00028 and sex: *P* = 0.36), **B**. PCoA plot using GUniFrac distance metrics (PERMANOVA, ANOSIM: diet: r2 = 0.18, *P* = 0.001, sex: r2 = 0.033, *P* = 0.64). Light red and dark red circles are females fed chow or HFD. Light blue and dark blue squares are males fed chow or HFD, **C**. Violin plot illustrating the 20 most abundant bacterial families present in the two diet-groups. Green represents chow fed pigs while yellow represent HFD fed pigs, and the black dots indicate the median values. The abundance is presented as percent on a log10 scale. The statistics correspond to DESeq2, DAtest package produced using R indicating which of these 20 most abundant bacterial families that were significantly different in abundance between the two diet groups. The asterisks indicate the statistical significance: * *P*<0.05, ** *P*<0.01, *** *P*<0.001.

To evaluate the effect of HFD feeding on the colon microbiota composition, standard 16S rRNA gene amplicon sequencing was carried out. Alpha-diversity in the colon microbiota was significantly lower in the HFD minipigs compared to chow whereas it was not impacted by sex (two-way ANOVA: diet: *P* = 0.00028 and sex: *P* = 0.36, Fig 3A). This was substantiated by a clear separation of colon microbiota from HFD- and chow-fed minipigs using GuniFrac similarity distance analysis (Adonis, diet: r2 = 0.18, *P* = 0.001, Fig 3B). Contrary to diet, sex did not influence the colonic microbiota as evaluated by GuniFrac distance analysis (Adonis, sex: *r*^*2*^ = 0.033, *P* = 0.64). Bacterial families contributing to the diet specific clustering of the colon microbiota based on significance in both the DESeq2 and the LEfSe analyses were *Clostridiaceae* (increased abundance with HFD), *Porphyromonadaceae*, *Fibrobacteraceae* and *Flavobacteriaceae* (all increased abundance with chow) (Fig 3C, S4A and S4B Table). In addition, in the DESeq2 analysis, *Sphingobacteriaceae* and *Streptococcaceae* were increased with chow diet, and *Enterobacteriaceae*, *Eubacteriaceae* and *Bacteroidaceae* were increased with HFD (Fig 3C, S4A and S4B Table). However, these families were not confirmed by the LEfSe analysis, where, on the other hand, *Acidaminococcaceae*, *Verrucomicrobiaceae*, *Spirochaetaceae* and *Prevotellaceae* were found to be significantly associated with chow diet (S4A and S4B Table). In depth analysis of bacterial differences on genus/species level using both DESeq2 and LEfSe analysis are shown in S5A and S5B Table. An overall summary of the bacterial families, genera and species found to significantly differ between diet groups both in the DESeq2 and the LEfSe analysis is given in Table 1.

**Table 1:** Bacterial taxa significantly different between treatment diets.

Family	Genus	Species	Associated with
***Fibrobacteraceae***	*Fibrobacter*	*Fibrobacter intestinalis*	Chow
***Flavobacteriaceae***	NS	NS	Chow
***Porphyromonadaceae***	NS	NS	Chow
*Acidaminococcaceae*	*Phascolarctobacterium*	*Phascolarctobacterium succinatutens*	Chow
*Erysipelotrichaceae*	*Holdemanella*	*Holdemanella biformis*	Chow
*Lachnospiraceae*	*Clostridium XlVa*	*Clostridium aerotolerans*	Chow
*Lachnospiraceae*	*Lachnospiracea incertae sedis*	*Eubacterium eligens*	Chow
*Prevotellaceae*	*Prevotella*	*Prevotella dentasini*	Chow
*Ruminococcaceae*	*Clostridium IV*	*Clostridium leptum*	Chow
*Ruminococcaceae*	*Clostridium IV*	*Eubacterium siraeum*	Chow
*Ruminococcaceae*	*Faecalibacterium*	*Faecalibacterium prausnitzii*	Chow
*Ruminococcaceae*	*Flavonifractor*	*Flavonifractor plautii*	Chow
*Ruminococcaceae*	*Intestinimonas*	*Intestinimonas butyriciproducens*	Chow
*Ruminococcaceae*	*Ruminococcus*	*Ruminococcus bromii*	Chow
*Spirochaetaceae*	*Treponema*	*Treponema berlinense*	Chow
*Spirochaetaceae*	*Treponema*	*Treponema bryantii*	Chow
*Lachnospiraceae*	*Clostridium XlVa*	NA	Chow
*Lachnospiraceae*	*Blautia*	NA	Chow
*Prevotellaceae*	*Prevotella*	NA	Chow
*Ruminococcaceae*	*Ruminococcus*	NA	Chow
***Clostridiaceae***	*Clostridium sensu stricto*	NA	HFD
*Acidaminococcaceae*	*Phascolarctobacterium*	*Phascolarctobacterium succinatutens*	HFD
*Erysipelotrichaceae*	*Turicibacter*	*Turicibacter sanguinis*	HFD
*Eubacteriaceae*	*Eubacterium*	*Eubacterium coprostanoligenes*	HFD
*Lachnospiraceae*	*Cellulosilyticum*	*Cellulosilyticum_ruminicola*	HFD
*Lactobacillaceae*	*Lactobacillus*	***Lactobacillus*** *equicursoris*	HFD
*Lactobacillaceae*	*Lactobacillus*	*Lactobacillus mucosae*	HFD
*Peptostreptococcaceae*	*Terrisporobacter*	*Terrisporobacter glycolicus*	HFD
*Lachnospiraceae*	*Cellulosilyticum*	NA	HFD
*Prevotellaceae*	*Prevotella*	NA	HFD
*Lachnospiraceae*	*Blautia*	NA	HFD

Taxonomic classifications at the family, genus, and species levels, revealing statistically significant distinctions (p<0.05) between dietary groups (chow or HFD) as determined through DESeq2 (S5A Table) and LEfSe (S5B Table) analyses, both corrected for multiple testing. The term "associated with" denotes the dietary group (Chow or HFD) in which the respective bacteria exhibited enrichment. Families highlighted in bold font attained statistical significance at the overarching family level with both analyses. "NS" denotes instances where statistical significance was not observed. NA: Not available

### High fat diet modulates the circulating metabolomic profile

**Fig 4:**
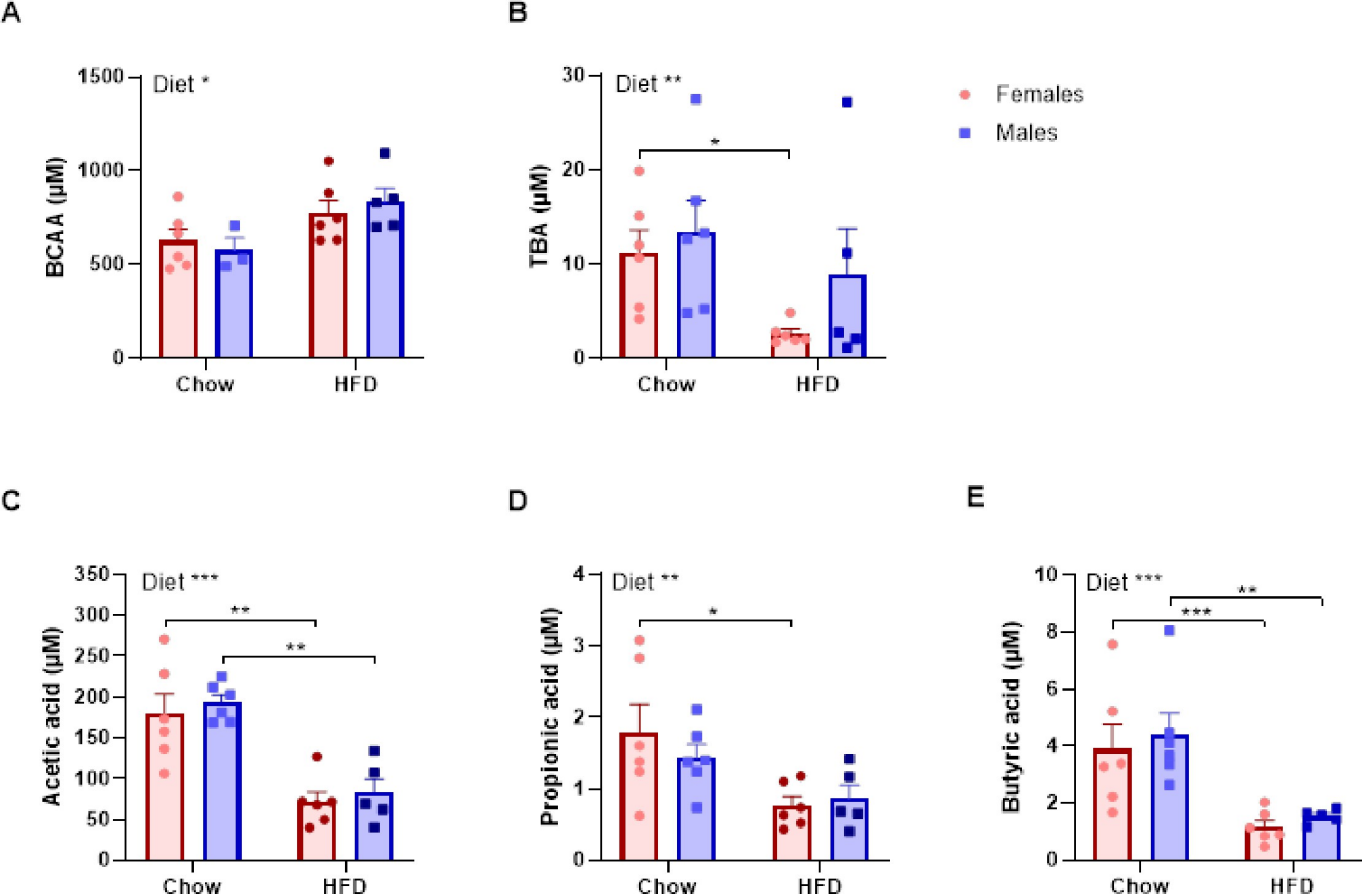
Metabolites and gut microbial products. Metabolites and gut microbial products in twenty-three female and male Göttingen Minipigs fed either chow or HFD for 12 weeks (n = 5–6). **A**. Branched chain amino acids (BCAA), **B**. Total bile acids (TBA), **C.** Acetic acid, **D.** Propionic acid and **E.** Butyric acid. Mean±SEM, n = 3–6 for BCAA and n = 5–6 for remaining parameters. *P<0.05, **P<0.01 and ***P<0.001 mark the level of statistical significance.

Significant changes in the gut microbial products were observed in the circulation. Acetic acid, propionic acid, butyric acid, isobutyric acid and isovaleric acid were all decreased in the HFD fed pigs compared to the chow fed pigs, whereas formic acid showed a small increase which was most pronounced in the male minipig (Fig 4C–4E, S3 Table).

HFD feeding led to moderately increased levels of BCAA, although only significant in the overall ANOVA analysis (Fig 4A and S3 Table). Similarly, there was a tendency for lower ImP with the HFD, although this was only statistically significant in the males (S3 Table).

**Fig 5:**
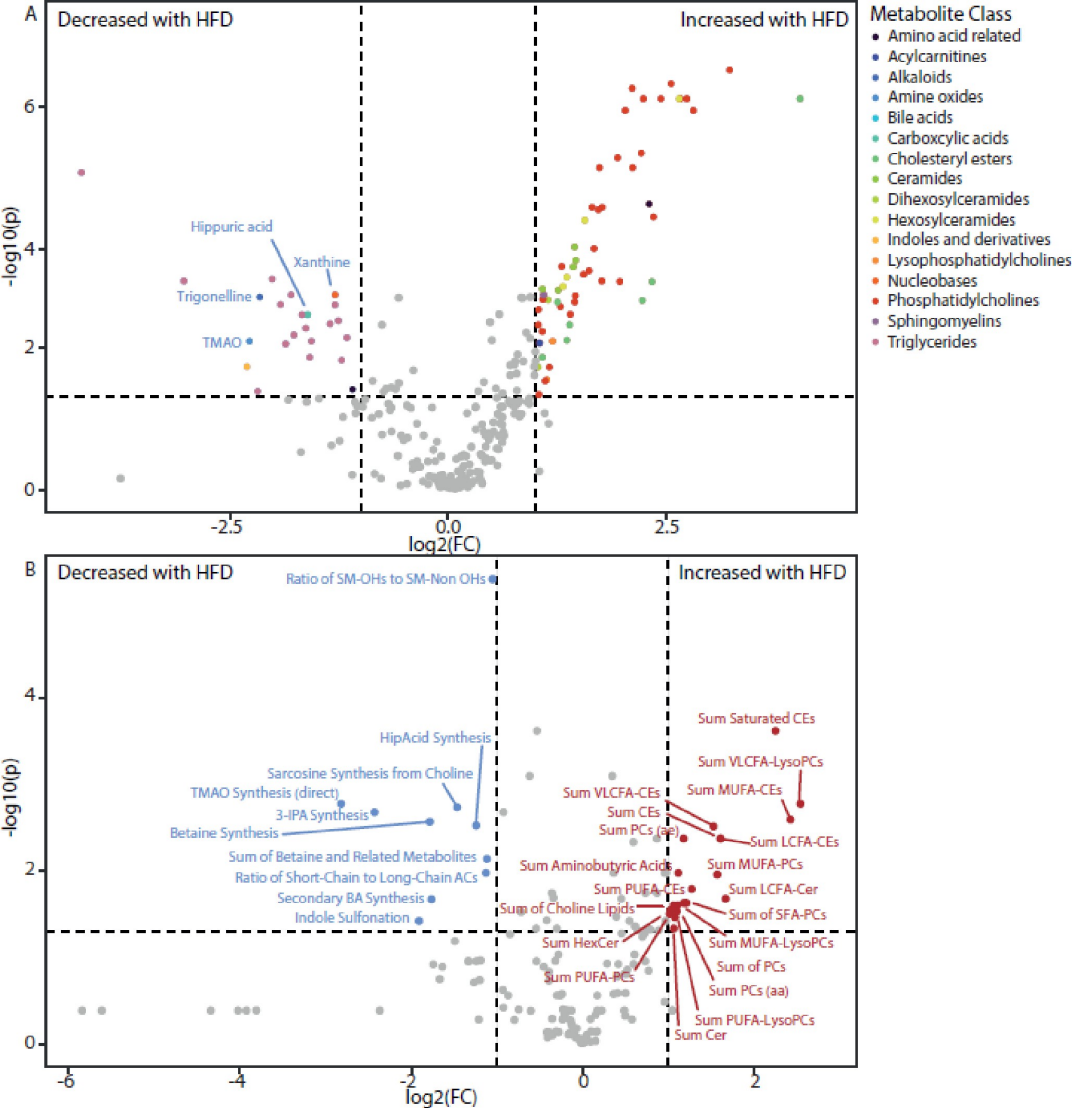
Plasma metabolic features. Volcano plots indicating the plasma metabolic features that significantly differ between high-fat diet-fed and chow-fed minipigs. This includes **A**. Individual metabolites (selected metabolites specified) and **B.** Metabolism indicators. Significant features must have a greater than two-fold difference between the groups in their abundance and an FDR-adjusted *P*<0.05. Colors indicate metabolic class. Individual features are presented with their fold change and significance in S6 Table.

Principal component analysis (PCA) of the metabolomic data showed clear separation between the plasma metabolic profiles from HFD-fed pigs and those fed chow, with no differences observed between females and males (S1A Fig). Similarly, the metabolic indicators clustered by feeding regime, although the separation was less distinct (S1B Fig). Comparing all minipigs that received the HFD with those on the chow diet identified 80 metabolic features to be altered (Fig 5A, S6 Table). As expected, most of these changes related to lipid metabolism with many phosphatidylcholines (PC), ceramides (Cer), hexosylceramides (HexCer), and dihexosylceramides increased with the HFD, as well as several cholesteryl esters (CE). Interestingly, 17 triglycerides were reduced after HFD feeding although no significant differences were noted in triglyceride-related metabolism indicators (*e*.*g*., sum of triglycerides, sum of saturated triglycerides, sum of unsaturated triglycerides, ratio of triglycerides to fatty acids; S2 Fig). Analysis of the metabolism indicator data revealed 2-5-fold increases in the sum of PCs, Cer, HexCer, CEs compared to the chow fed pigs (Fig 5B). In addition, the sum of choline lipids, and aminobutyric acids were also increased. The ratio of short-chain to long-chain acylcarnitines was reduced with HFD, as was the ratio of hydroxylated-sphingomyelins to non-hydroxylated sphingomyelins. Betaine (trimethylglycine) synthesis, sarcosine (methylglycine) synthesis from choline, and the sum of betaine and related metabolites were reduced with HFD, possibly reflecting the increased need for choline to synthesize lipoproteins for lipid export from the liver. Consistent with the observed reduction in alpha-diversity, several metabolites related to gut microbial activity were reduced following the HFD. This included a reduction in circulating hippuric acid, 3-indole-propionate (3-IPA), xanthine, trigonelline and trimethylamine *N*-oxide (TMAO) (Fig 5A, S6 Table). These changes were supported by the metabolism indicator data showing that TMAO, 3-IPA and hippurate synthesis were all reduced with HFD feeding, as well as secondary bile acid synthesis (Fig 5B, S6 Table).

Considering only the female minipigs, additional metabolic changes were observed between HFD-fed and the chow-fed minipigs (S3A Fig). A total of 113 features were altered with additional reductions in the microbial-derived metabolites of phenylalanine, phenylacetylglycine (PAG), and lysine, 5-aminovalerate (5AVA), and the secondary bile acid, glycolithocholic acid-sulfate (S7 Table). Fewer alterations were seen in the male minipigs associated with diet (21 features, S3B Fig, S8 Table), although this likely relate to the small group sizes in this dataset (HFD *n* = 5, Chow *n* = 3).

### Differential gene expression analysis in liver, fat and muscle

Biomark platform: After manual curation and processing of qPCR data, 64 assays were accepted for further analysis in liver, 56 in omentum and 52 in skeletal muscle (S2 Table) out of 96 profiled gene-assays. Beta-actin (ACTB), TATA box binding protein (TBP), & tyrosine 3-monooxygenase/tryptophan 5-monooxygenase activation protein zeta (YWHAZ), were chosen as reference genes for liver data, ACTB and YWHAZ were chosen as reference genes for omentum data and TBP and ribosomal protein L4 (RPL4) were chosen as reference genes for skeletal muscle data.

Mx3005P platform: BAAT gene was not expressed in the liver, GPBAR1 assay was not specific, and hence five genes were accepted for further analysis in liver (S2 Table). ACTB, TBP and YWHAZ were used for normalization.

Differential expression analysis in liver revealed 6 genes significantly differentially expressed (DE) after MTC between chow diet and HFD with a fold-change (FC)>2 or <-1.5. Genes involved in triglyceride synthesis (*LPIN1*), cholesterol metabolism (*FDFT1*, *LDLR*), lipogenesis (*IGFBP2*) and adipogenesis (*KLB*) had decreased expression while a gene involved in fatty acid oxidation (*CPT1A*) had elevated expression in HFD compared to chow fed minipigs (Table 2). In the omental adipose tissue, 9 genes were significantly DE after MTC between HFD and chow fed animals with FC>2 or <-1.5. Gene expression for satiety hormone leptin (*LEP*) and genes involved in synthesis of mono-unsaturated fatty acids (*SCD*), fatty acid synthesis (*ACACA*), fatty acid metabolism (*UCP3*), adipose tissue metabolism (*AGT*), triglyceride metabolism and fatty acid oxidation (*PPARA*) and cholesterol metabolism, lipogenesis and glucose homeostasis (*INSIG1*) had increased gene expression in HFD fed minipigs compared to chow-fed minipigs (Table 2). The expression of genes *GRB10* and *TIMP1*, inhibitors of insulin and adipocyte differentiation, respectively, was decreased in omentum of HFD fed minipigs (Table 2). We did not find any significantly DE genes in skeletal muscle tissue between diets when applying the FC>2 or <-1.5 and MTC (S2 Table). Moreover, we did not find any significantly DE gene after MTC when comparing the two sexes.

**Table 2:** Differentially expressed genes in liver and omental adipose tissue.

Tissue	Gene	FC	↑/↓ in HFD	P-Value	Pathway
Liver	*LPIN1*	-2.04	**↓**	>0.00001	Triglyceride synthesis
	*FDFT1*	-2.25	**↓**	0.00020	Cholesterol biosynthesis
	*IGFBP2*	-3.06	**↓**	>0.00001	Lipogenesis
	KLB	-4.11	**↓**	0.00016	Regulator of adipogenesis
	LDLR	-2.44	**↓**	0.00047	Cholesterol uptake
	*CPT1A*	2.93	**↑**	>0.00001	Long-chain fatty acid (FA) oxidation
Omental adipose tissue	*GRB10*	-1.63	**↓**	0.00022	Inhibitor of insulin
	*TIMP1*	-1.80	**↓**	0.00089	Inhibits adipocyte differentiation
	*LEP*	50.66	**↑**	>0.00001	Satiety hormone
	*SCD*	24.61	**↑**	>0.00001	Synthesis of mono-unsaturated FA
	*ACACA*	3.52	**↑**	0.00014	FA synthesis
	*UCP3*	2.63	**↑**	0.00045	FA metabolism
	*AGT*	2.48	**↑**	0.00022	Adipose tissue metabolism and whole-body metabolism.
	*INSIG1*	2.14	**↑**	0.00066	Regulates cholesterol metabolism, lipogenesis, and glucose homeostasis
	*PPARA*	2.00	**↑**	0.00028	Lipoprotein & triglyceride metabolism, FA oxidation, FA uptake & export

Significantly differentially expressed (DE) genes after multiple test correction (MTC) with Fold change (FC) >2 or <-1.5 in liver and omental adipose tissue from lean and obese castrated male and ovariectomized female Göttingen Minipigs. N = 5–6. Arrows indicate decreased (↓) or increased (↑) gene expression in the HFD groups. T-test followed by MTC was applied to compare the diet groups as there were not differences between sexes. The last column shows in which pathway the gene is involved.

### High fat diet induced significant fat cell hypertrophy and glomerular vacuolation

Organ weights (absolute and relative to BW and lean mass) are shown in S9 Table. Absolute liver, heart and kidney weights were all significantly increased and relative heart weight was significantly decreased in the HFD-fed compared to chow-fed animals. In the kidney of all animals fed HFD and one animal from the female chow group moderately enlarged glomeruli were observed (S4 Fig). The glomeruli presented varying degrees of vacuolation and occasional minor interstitial infiltration of mainly mononuclear cells. In addition, Bowman`s space was often enlarged and in several of the animals this vacuolation could also be observed in a few and occasionally several tubuli. The changes were not further characterised but could represent lipid as previously described [36]. Moreover, mild, diffuse hypertrophy of adipocytes were recorded in all examined white adipose tissue (WAT) depots form the HFD fed animals (both males and females) (S4 Fig).

### Clinical chemistry and serum/plasma biomarkers

Clinical chemistry and serum/plasma biomarkers can be found in S3 Table.

In brief, in addition to the biomarker changes described above, the following overall observations were made: AST, ALT, ALP, creatinine and sodium were significantly lower, while glycerol and haptoglobin were significantly higher in the HFD groups compared to chow. No effect of diet or sex was observed in the remaining parameters (S3 Table).

## Discussion

**Fig 6:**
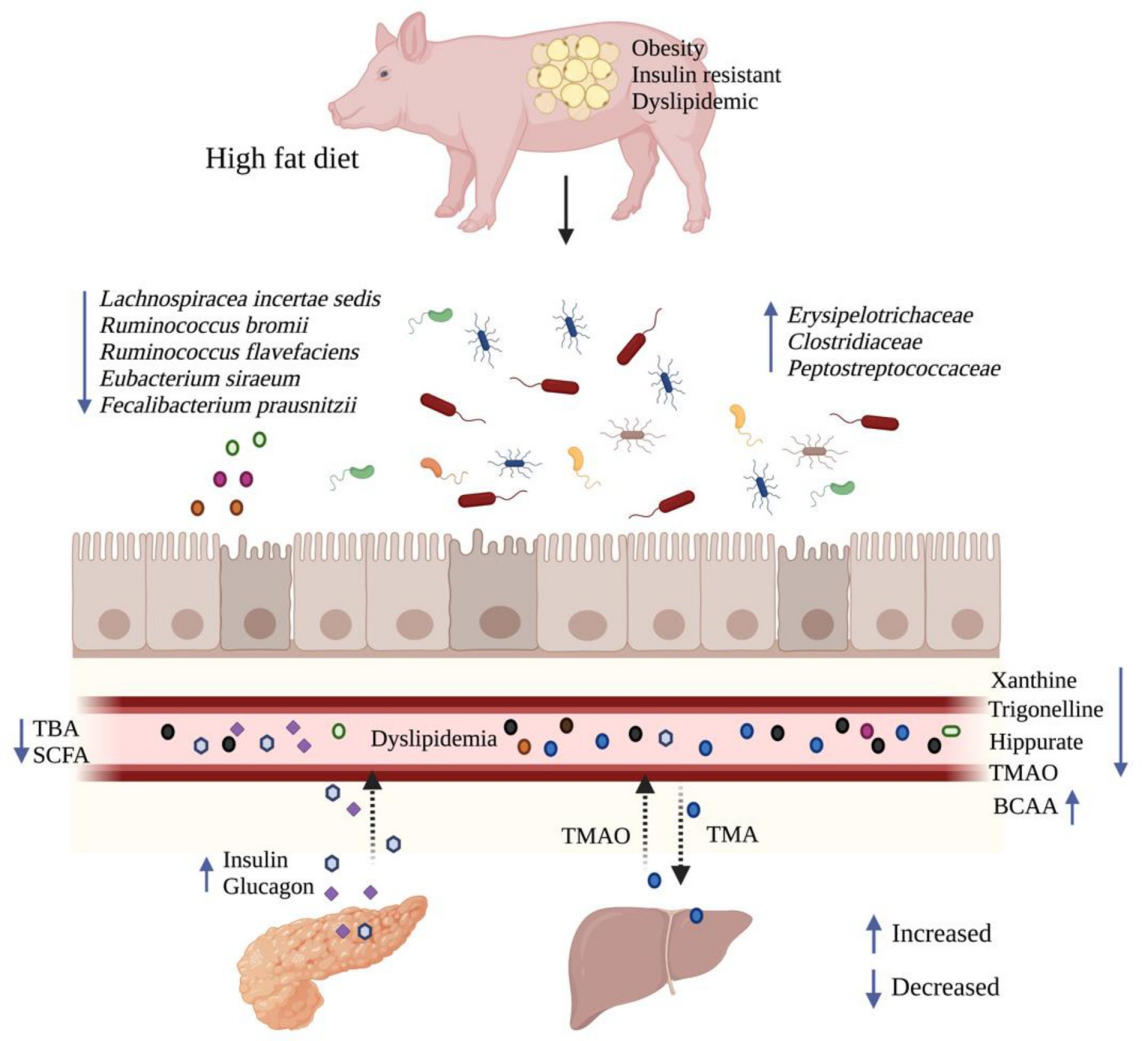
Graphical overview. HFD caused obesity, dyslipidemia, insulin resistance and increased circulating levels of fasting insulin and glucagon. Moreover, HFD feeding was followed by an increase in relative abundance of bacterial families *Erysipelotrichaceae*, *Clostridiaceae* and *Peptostreptococcaceae* in the colon microbiota and a decrease in circulating levels of hippuric acid, xanthine, trigonelline, TMAO, SCFA and TBA in addition to an increase in branched-chain amino acids (BCAA). Arrows ↓ and ↑ indicate a decrease or an increase in a measured parameter, respectively. BCAA: branched chain amino acids, HFD: high fat diet, SCFA: short chain fatty acids, TBA: total bile acids, TMAO: trimethylamine N-Oxide. Created with biorender.com.

Overall, significant obesity and components of the metabolic syndrome were induced with only 3 months of HFD-feeding in young growing OVX or castrated Göttingen Minipigs. The obese pigs were characterized by dyslipidemia and insulin resistance, and significant diet specific clustering of the colon microbiota was mainly attributed to changes in bacterial families *Clostridiaceae*, *Porphyromonadaceae*, *Fibrobacteraceae and Flavobacteriaceae*. Consistent with these changes in the colon microbiota several products of gut microbial metabolism that have previously been linked with a positive effect on metabolism and health were reduced in the circulation, e.g. hippurate, trigonelline and xanthine (Fig 6). These changes may have contributed to the impaired metabolic phenotype in the minipig model.

Twelve weeks of HFD significantly increased the BW and fat percentage compared to chow fed minipigs, leading to a severe, human-relevant level of obesity in both castrated male and OVX female minipigs. The sex differences previously observed in obesity development in intact, young Göttingen Minipigs [37] are thus seemingly abolished by neutering. The increased fat percentage is likely the main reason for the highly upregulated gene expression level of leptin in omental adipose tissue, as observed previously in both obese humans and pigs [38–40]. However, the approx. 50-fold increase we observed in the HFD-fed Göttingen Minipigs is remarkable and may indicate an even greater degree of leptin resistance than observed in humans [41] and/or a very low level in the chow-fed animals that were fed restrictedly. It has previously been reported that the obesity-prone Iberian pig has a polymorphism of the leptin receptor gene which affects leptin sensitivity [42], and it would be interesting to study if this is also the case in Göttingen Minipigs.

In addition, genes involved in fatty acid synthesis and metabolism (e.g. *SCD*, *ACACA*, *UCP3*, *AGT and PPARA*) were upregulated in the omentum in the HFD-fed state, whereas *GRB10* gene, an inhibitor of insulin signaling, was decreased. It has been shown that fat-specific disruption of *GRB10* gene increases mTORC1 signaling in adipose tissues, suppresses lipolysis, and reduces thermogenic function, thus enhancing fat deposition [43]. Nevertheless, profiling *mTOR* gene in liver did not show DE between the two diet or sex groups.

With respect to plasma lipids, HFD feeding increased total serum cholesterol and decreased HDL/non-HDL-cholesterol ratio, indicating dyslipidemia. As a consequence of the increased plasma cholesterol levels in the HFD groups, hepatic expression of *LDLR* and *FDFT1*, catalyzers of the first step in cholesterol biosynthesis pathway, was decreased, which may be a rescue mechanism in order to lower hepatic cholesterol accumulation [44, 45]. Likewise, regulators of TG synthesis and lipogenesis such as *LPIN1*, *KLB* and *IGFB2* genes were downregulated in the liver of the HFD-fed minipigs, whereas *CPT1A* gene was upregulated, indicating increased fatty acid oxidation (Table 2). The decrease in *LDLR*, *FDFT1* and *KLB* genes has also been observed previously in Göttingen Minipigs fed a cholesterol and fat-rich diet [46].

Circulating total TG was similar in the HFD and the chow fed pigs, but there were significant changes in individual TG species caused by the HFD. Of the 185 TG species measured, 28 were decreased by the HFD in the females (17 when both sexes considered) whereas the remaining were unchanged (Fig 5). *Faecalibacterium prausnitzii* and *Eubacterium Eligens*, that are both more abundant in the chow-fed minipigs relative to the HFD fed minipigs in the present study, have been shown to be negatively associated with TG in humans [47, 48]. In addition, the serum lipid profile had increased levels of the species C3-DC, CE, Cer, HexCer, LysoPC and PC. Increases in both Cer, LysoPC, PC and CE are linked to human obesity, insulin resistance and low-grade inflammation [49–52]. Cer antagonize insulin signaling through inhibition of transmission signals of phosphatidylinositol-3 kinase (PI3K) thereby blocking the activation of anabolic enzyme Akt/PKB [49]. In this manner, Cer interfere with glucose uptake, and decreased plasma Cer has been shown to improve insulin signaling [49].

Minimal modeling of IVGTT data showed no significant effect of HFD on SI, but SG was significantly decreased in HFD minipigs indicating a decreased ability of glucose to suppress endogenous glucose production and/or decreased ability to stimulate peripheral glucose uptake [53]. This decrease in SG is consistent with the situation in humans, where SG decreases both in obese and type 2 diabetic individuals [54, 55]. Both HOMA-IR and Matsudas insulin sensitivity index indicated increased insulin resistance in the HFD fed minipigs, an effect that was most pronounced in the OVX female minipigs based on the post hoc analysis. An increased propensity in females to develop insulin resistance has previously been described in intact Göttingen Minipigs [37] as well as in humans [56]. Furthermore, circulating haptoglobin, independently associated with hyperinsulinemia [57], was significantly elevated in the HFD fed minipigs (S3 Table), but the deregulation of the *HP* transcript only showed a very modest fold change which did not reach statistical significance. Lastly, the fasting glucagon was also significantly higher in the HFD-fed minipigs compared to chow-fed, whereas the glucagon receptor gene expression in the liver was not significantly changed. The altered glucagon regulation may be caused by insulin resistant alpha cells or by the overall higher protein/food intake in the HFD-fed animals and may over time lead to poor glycemic control and prediabetes as observed in humans [58–61].

Several of the bacterial genera and species that were changed with the HFD feeding in the present study have been implicated in both obesity and/or insulin resistance and type 2 diabetes in humans. *Faecalibacterium prausnitzii*, *Eubacterium siraeum*, *Eubacterium Eligens* and *Ruminococcus bromii* (Table 1, S5A and S5B Table) showed lower relative abundance in HFD fed pigs, which is in agreement with studies in overweight or obese subjects [48, 62–65]. In addition, *Faecalibacterium prausnitzii* has been found to be more abundant in the gut of individuals with normal glucose tolerance compared to individuals with type 2 diabetes [66, 67]. Members of the *Clostridiaceae* (*Clostridium sensu stricto*) family have previously been linked to an obese phenotype [68], and members of *Peptostreptococcaceae* have similarly been identified with a three-fold increase in obese children compared to normal weight [69]. *Clostridiaceae* and members of bacterial family *Peptostreptococcaceae* had higher relative abundance in the HFD-fed compared to the chow-fed minipigs (Table 1, S4 Table). In diet-induced obese mice, an increase in taxa belonging to *Clostridiaceae* and *Peptostreptococcaceae* have also been associated with impaired glucose tolerance and impaired insulin clearance [70]. However, reduction in relative abundance of *Clostridiaceae* has also been reported with the onset of childhood obesity (age 2–7 year) and in humans with obesity [71], underlining that different members of this family likely influence glucose metabolism differently. Genus *Blautia* was found to be associated both with HFD and chow diet which is in line with the both higher and lower relative abundance of this genus observed in obese vs. non-obese persons [72]. Other studies support a protective role of the *Blautia* genus in obesity and type 2 diabetes, with this genus being inversely correlated to visceral fat mass [73], and the odds ratios of obesity and type 2 diabetes in Japanese subjects [74].

Several circulating factors related to the gut microbiota were modulated by the diet. The amount of circulating BCAA were elevated in the HFD minipigs (Fig 4), which has been associated with obesity, insulin resistance and type 2 diabetes [75]. A decrease in gut bacteria responsible for BCAA uptake and catabolism, such as *Butyrivibrio crossotus* and *Eubacterium siraeum*, may lead to BCAA accumulation and have been linked to insulin resistance [75]. In line with this, the colon microbiota of HFD minipigs showed reduced relative abundance of *Eubacterium siraeum* (Table 1, S5A and S5B Table). Increased amounts of BCAAs have been shown to cause persistent activation of hepatic protein complex mTORC1 affecting insulin signaling in the liver [9, 10]. However, at gene level *mTORC1* in liver was not regulated by the diet and there were no significant changes in the insulin signaling pathways in the liver apart from a slight decrease in the expression of the insulin receptor and insulin receptor substrate 2 (S2 Table), indicating that the moderate increase in BCAA observed here was not clinically relevant.

The gut microbial metabolite hippurate, which is associated with increased microbial diversity and reduced risk of having metabolic syndrome [76], was significantly decreased in the HFD groups compared to chow groups, which may have contributed to the metabolic changes observed. Hippurate is associated with increased abundance of OTUs identified as *Fecalibacterium prausnitzii* [76], which despite being present at low relative abundance (mean relative abundance: Chow 0.11%; HFD 0.051%) in this study, was indeed found to be decreased in relative abundance in the pigs exposed to HFD (Table 1, S5A and S5B Table).

Hippurate has also been described as negatively associated with genera *Eubacterium* and *Ruminococcus* [76], and in line with this we observed an increase in OTUs belonging to the *Eubacterium* genus in the HFD fed pigs. However, this was not the case for the *Ruminococcus* genus that was enriched in the chow-fed pigs (S5A and S5B Table). Discrepant results for *Ruminococcus* and several other genera have also been found in human studies as indicated in a recent meta-analysis comparing the gut microbiome composition in obese and non-obese persons [72], suggesting that different strains of the same species/genus might be differentially associated with different dietary components /metabolic traits.

Moreover, HFD significantly decreased the circulating level of TMAO, that is produced in the liver from the gut microbial metabolite trimethylamine (TMA), a product of choline degradation. The hepatic expression of Flavin Containing Dimethylaniline Monoxygenase 3 (FMO3) that converts TMA to TMAO was not regulated by HFD and the most likely explanation for the decreased levels of TMAO is thus decreased microbial production of TMA. TMAO has been associated with attenuated ER stress, reduced lipogenesis in adipocytes, improved insulin secretion and restored glucose tolerance in mice [77]. Others report a causative role for TMAO in cardiovascular disease and non-alcoholic steatohepatitis [78], indicating that more studies are needed to further elucidate the effects of this metabolite. Another gut microbial metabolite, trigonelline, was also found to be decreased by HFD in the current study, which is in accordance with observations in humans where an unhealthy plant-based diet decreased trigonelline compared to a healthy plant-based diet [79]. In diabetic rats, treatment with trigonelline reduced oxidative stress and protected β‐cells from glucotoxicity [80]. Similarly, circulating xanthine was reduced in the HFD-fed minipigs, which suggests a significantly higher activity of xanthine oxidase, the rate-limiting enzyme in the conversion of xanthine to uric acid. This hypothesis is supported by observations in obese humans where lower urinary levels of xanthine are observed [81] and where xanthine oxidase activity is independently and positively associated with obesity, insulin resistance and uric acid concentrations [82].

Interestingly, the gut microbial metabolite ImP, previously associated with type-2 diabetic patients [83], was significantly lower in castrated males on HFD compared to chow, whereas no effect of diet was observed in the OVX female minipigs (S3 Table). The fact that ImP was not increased in these obese, insulin resistant minipigs is in line with data from obese, non-diabetic subjects, where ImP levels only correlated to blood pressure but not insulin resistance [84].

In addition, serum levels of SCFAs were overall decreased in HFD fed animals (Fig 4), which may be a result of the much lower fiber content in the HFD compared to the chow leading to a lower abundance of bacteria species involved in SCFA production, e.g. *Faecalibacterium prausnitzii*, *Fibrobacter intestinalis*, *Ruminococcus bromii* and *Phascolarctobacterium succinatutens* [85–88]. The signaling pathways and metabolic effects of SCFAs has been reviewed by He et al. [8]. Briefly, SCFA exert their actions on target cells through free fatty acid receptors 2 and 3 (FFAR2/3). These receptors are expressed in many different cell types in the host including adipose tissue, liver, skeletal muscle and pancreas where their activation by SCFA leads to beneficial effects on glucose and lipid metabolism in addition to anti-inflammatory effects [8]. In addition, acetate has been involved in appetite regulation through central mechanisms [89], and the lower levels of SCFA in the HFD-fed pigs may thus lead to further increased appetite, obesity and adverse metabolic effects.

When assessing the serum BA species, we found that HFD reduced TBA and lead to a reduction in secondary BA synthesis in the female HFD-fed minipigs, with specific reduction in the circulating amount of the conjugated secondary BA, glycolithocholic acid-sulfate (Fig 5A, S7 Table). However, it must be noted that the BA measured in this study did not include the pig specific HCA and HCDA which account for 40–90% of the plasma BA pool in Göttingen Minipigs [90, 91]. As such, these observations should be treated with caution.

Taken together, feeding a caloric excess of a HFD diet compared to feeding chow-diet restrictedly was associated with a dysbiotic phenotype in the colon, comparable to what is observed with human obesity/metabolic syndrome. These microbial changes and the following changes in microbial products might have contributed to the impaired metabolic health of these minipigs. The young HFD fed minipig thus represents an interesting translational obesity model, and at the same time it is also smaller, easier to handle and less prone to overload-induced lameness compared to previously described obesity models, where the pigs typically weighed 80–100 kg [16, 22].

This paper focused on the association between the metabolic phenotype and the microbial composition and their products, and the data and hypotheses generated here could inspire for additional cellular and molecular experiments in future dedicated experiments.

## Conclusion

In summary, feeding a HFD for only 3 months induced marked obesity with impairments of glucose and lipid metabolism in young, castrated male and ovariectomized female Göttingen Minipigs. Furthermore, HFD-feeding led to significant changes in the colonic gut microbiota and in gut microbial products, which likely contributed to the impaired metabolic phenotype observed in this minipig model (Fig 6). No significant differences between the two sexes were observed in the measured microbial, metabolic or gene expression parameters in these neutered animals, although the metabolic phenotype seemed to be slightly more pronounced in the OVX females. Many of the metabolic, gene expression and gut microbial changes are consistent with observations in humans living with obesity, making the young, obese Göttingen Minipig a relevant alternative model for studying the complex interplay between the host metabolism and gut microbiota and its products.

## Supporting information

S1 File Supplementary methods description. (PDF)

S1 Fig Principal components analysis on the plasma metabolomics data. Scores plots from the principal components analysis (PCA) model constructed on the plasma (A) metabolic profiles and (B) metabolism indicators from high-fat diet (HFD)-fed minipigs and those receiving chow.
(TIF)

S2 Fig Box plots showing triglyceride-related metabolism indicators across the study groups. (TIF)

S3 Fig Volcano plots indicating the plasma metabolic features that significantly differ between high-fat diet-fed and chow-fed minipigs stratified by sex. Metabolites compared between **A.** Female HFD-fed and chow-fed minipigs and **B.** Male HFD-fed and chow-fed minipigs. **C.** Metabolism indicators compared between Female HFD versus chow. No metabolism indicators were found to significantly differ in the males based on HFD feeding. Significant features must have a greater than two-fold difference between the groups in their abundance and an FDR-adjusted p < 0.05. Colors indicate metabolic class.
(TIF)

S4 Fig Histological findings in adipose tissue and kidneys. A) Normal size kidney glomerulus from a control female Göttingen Minipig, B) Moderately enlarged kidney glomerulus from a DIO female Göttingen Minipig, C) Normal size subcutaneous adipocytes from a control female Göttingen Minipig, D) Mildly hypertrophied subcutaneous adipocytes from a DIO female Göttingen Minipig.
(TIF)

S1 Table Diet specifications. (DOCX)

S2 Table Gene expression data. (XLSX)

S3 Table Clinical chemistry and serum/plasma biomarkers. (DOCX)

S4 Table Bacterial taxa at family level significantly different between test diets having sex as cofactor. **A.** Bacterial taxa at family level significantly different between test diets having sex as cofactor. DESeq2 wrapped in DAtest package was used. Table includes bacterial families with log Fold Change above 2 or below -2 and an adjusted p-value < 0.05 was considered significantly different between the groups. OUT: operational taxonomic units, HFD: high fat diet. **B.** Bacterial taxa at family level significantly different between treatment diets. Diet is included as class and sex as subclass using Huttenhower LEfSe online tool with default settings. Kruskal-Wallis test for classes reached significance when p < 0.05 and 2.5 for threshold on the logarithmic LDA score for discriminative features. Picture exported from https://huttenhower.sph.harvard.edu/galaxy/.
(DOCX)

S5 Table Bacterial taxa were significantly different between diets having sex as covariate. **A.** Bacterial taxa significantly different between treatment diets having sex as covariate and mapped to taxonomical level genus or species. DESeq2 wrapped in DAtest r package was used. Table includes log Fold Change above 2 or below -2. OTUs mapped at species level and an adjusted p-value < 0.05 was considered significantly different. HFD: high fat diet. **B.** Significantly different OTU between treatment diets. Diet is included as class and sex as subclass using Huttenhower LEfSe online tool with default settings. Kruskal-Wallis test for classes and 2.5 for threshold on the logarithmic LDA score for discriminative features. Picture exported from https://huttenhower.sph.harvard.edu/galaxy/ and has been split into 5 images separate images (indicated in each split figure in the top right corner—x/5).
(DOCX)

S6 Table Plasma metabolites that significantly differ between HFD-fed and chow-fed minipigs. Significant features have a greater than two-fold difference between the study groups and an FDR-adjusted p < 0.05, calculated by a Student’s t-test.
(XLSX)

S7 Table Plasma metabolites that significantly differ between female HFD-fed and chow-fed minipigs. Significant features have a greater than two-fold difference between the study groups and an FDR-adjusted p < 0.05, calculated by a Student’s t-test.
(XLSX)

S8 Table Plasma metabolites that significantly differ between male HFD-fed and chow-fed minipigs. Significant features have a greater than two-fold difference between the study groups and an FDR-adjusted p < 0.05, calculated by a Student’s t-test.
(XLSX)

S9 Table Body composition and organ weights. Body composition data and absolute and relative organ weights in castrated male (M) and ovariectomised female (F) Göttingen Minipigs fed chow or high fat diet (HFD). Two-way ANOVA with gender and diet as explanatory variables followed by Tukeys multiple comparison test. Only the following comparisons were considered relevant: M-HFD vs. M-chow, M-HFD vs. F-HFD, M-chow vs. F-chow and F-HFD vs. F-chow. * p<0.05, ** p<0.01, *** p<0.001.
(DOCX)

## Abbreviations

ACACA: Acetyl-CoA Carboxylase Alpha
ACTB: Beta-actin
AGT: Angiotensinogen
AUC: area under the curve
BA: Bile acids
BAAT: bile acid
CoA: amino acid N-acyltransferase
BCAA: branched chain amino acids
BW: Body weight
C3-DC: hydroxybutyrylcarnitine
CE: cholesteryl esters
Cer: ceramides
CPT1A: Carnitine Palmitoyltransferase 1A
FC: fold change
FDFT1: Farnesyl-diphosphate Farnesyltransferase 1
FDR: false discovery rate
FGF19: fibroblast growth factor-19
FMO3: Flavin Containing Dimethylaniline Monoxygenase 3
FXR: farnesoid X receptor
GLCAS: glycine conjugated secondary bile acid
GPBAR1: G Protein-Coupled Bile Acid Receptor 1
GRB10: Growth Factor Receptor Bound Protein 10
HDL: : high-density lipoprotein
HexCer: hexosylceramides
HFD: high fat diet
IGFBP2: Insulin-like Growth Factor-binding Protein 2
ImP: Imidazole Propionate
INSIG1: : Insulin-induced Gene 1 Protein
IVGTT: : Intravenous glucose tolerance test
KLB: Beta-Klotho
LDA: linear discriminant analysis
LDL: : low density lipoprotein
LDLR: Low Density Lipoprotein Receptor
LEfSe: linear discriminant analysis effect size
LEP: Leptin
LPIN1: Lipin 1
LysoPC: lysophosphatidylcholines
MMT: Mixed meal test
MTC: Bonferroni’s multiple test correction
mTORC1: mammalian target of rapamycin complex 1
NextSeq: Illumina Next generation sequencing based
OTUs: operational taxonomic Units
OVX: ovariectomized
PC: phosphatidylcholines
PCA: Principal component analysis
PPARA: Peroxisome Proliferator Activated Receptor Alpha
RPL4: ribosomal protein L4
SCD: Stearoyl-CoA Desaturase 1
SCFA: short chain fatty acids
SG: glucose clearance
SI: insulin sensitivity index
TBA: total bile acids
TBP: TATA box binding protein
TG: triglycerides
TGR5: Takeda G protein-coupled receptor 5
TIMP1: Metalloproteinase Inhibitor 1
TMA: trimethylamine
TMAO: trimethylamine N-Oxide
UCP3: Uncoupling Protein 3
WAT: : White adipose tissue
YWHAZ: : tyrosine 3-monooxygenase/tryptophan 5-monooxygenase activation protein zeta

## References

[1] WHO. *Obese and overweight* 2021 [updated 2021.08.26]. Available from: [https://www.who.int/news-room/fact-sheets/detail/obesity-and-overweight].

[2] Cook S, Kavey RE. Dyslipidemia and pediatric obesity. Pediatr Clin North Am. 2011;58(6):1363–73, ix. Epub 2011/11/19. doi: 10.1016/j.pcl.2011.09.003 ; PubMed Central PMCID: PMC3220879. 22093856 PMC3220879

[3] Klop B, Elte JW, Cabezas MC. Dyslipidemia in obesity: mechanisms and potential targets. Nutrients. 2013;5(4):1218–40. Epub 2013/04/16. doi: 10.3390/nu5041218 ; PubMed Central PMCID: PMC3705344. 23584084 PMC3705344

[4] Davis CD. The Gut Microbiome and Its Role in Obesity. Nutr Today. 2016;51(4):167–74. Epub 2016/11/01. doi: 10.1097/NT.0000000000000167 ; PubMed Central PMCID: PMC5082693. 27795585 PMC5082693

[5] Fan Y, Pedersen O. Gut microbiota in human metabolic health and disease. Nat Rev Microbiol. 2021;19(1):55–71. Epub 2020/09/06. doi: 10.1038/s41579-020-0433-9 . 32887946

[6] Rampelli S, Guenther K, Turroni S, Wolters M, Veidebaum T, Kourides Y, et al. Pre-obese children’s dysbiotic gut microbiome and unhealthy diets may predict the development of obesity. Commun Biol. 2018;1:222. Epub 2018/12/12. doi: 10.1038/s42003-018-0221-5 ; PubMed Central PMCID: PMC6286349. 30534614 PMC6286349

[7] Aoun A, Darwish F, Hamod N. The Influence of the Gut Microbiome on Obesity in Adults and the Role of Probiotics, Prebiotics, and Synbiotics for Weight Loss. Prev Nutr Food Sci. 2020;25(2):113–23. Epub 2020/07/18. doi: 10.3746/pnf.2020.25.2.113 ; PubMed Central PMCID: PMC7333005. 32676461 PMC7333005

[8] He J, Zhang P, Shen L, Niu L, Tan Y, Chen L, et al. Short-Chain Fatty Acids and Their Association with Signalling Pathways in Inflammation, Glucose and Lipid Metabolism. Int J Mol Sci. 2020;21(17). Epub 2020/09/06. doi: 10.3390/ijms21176356 ; PubMed Central PMCID: PMC7503625. 32887215 PMC7503625

[9] Yoon MS. The Emerging Role of Branched-Chain Amino Acids in Insulin Resistance and Metabolism. Nutrients. 2016;8(7). Epub 2016/07/05. doi: 10.3390/nu8070405 ; PubMed Central PMCID: PMC4963881. 27376324 PMC4963881

[10] Gojda J, Cahova M. Gut Microbiota as the Link between Elevated BCAA Serum Levels and Insulin Resistance. Biomolecules. 2021;11(10). Epub 2021/10/24. doi: 10.3390/biom11101414 ; PubMed Central PMCID: PMC8533624. 34680047 PMC8533624

[11] Moran-Ramos S, Macias-Kauffer L, López-Contreras BE, Villamil-Ramírez H, Ocampo-Medina E, León-Mimila P, et al. A higher bacterial inward BCAA transport driven by Faecalibacterium prausnitzii is associated with lower serum levels of BCAA in early adolescents. Mol Med. 2021;27(1):108. Epub 2021/09/17. doi: 10.1186/s10020-021-00371-7 ; PubMed Central PMCID: PMC8444488. 34525937 PMC8444488

[12] Zhang S, Zeng X, Ren M, Mao X, Qiao S. Novel metabolic and physiological functions of branched chain amino acids: a review. J Anim Sci Biotechnol. 2017;8:10. Epub 2017/01/28. doi: 10.1186/s40104-016-0139-z ; PubMed Central PMCID: PMC5260006. 28127425 PMC5260006

[13] Bollen P, Ellegaard L. The Göttingen minipig in pharmacology and toxicology. Pharmacol Toxicol. 1997;80 Suppl 2:3–4. Epub 1997/01/01. doi: 10.1111/j.1600-0773.1997.tb01980.x . 9249853

[14] Koopmans SJ, Schuurman T. Considerations on pig models for appetite, metabolic syndrome and obese type 2 diabetes: From food intake to metabolic disease. Eur J Pharmacol. 2015;759:231–9. Epub 2015/03/31. doi: 10.1016/j.ejphar.2015.03.044 . 25814261

[15] Larsen MO, Rolin B, Wilken M, Carr RD, Svendsen O. High-fat high-energy feeding impairs fasting glucose and increases fasting insulin levels in the Gottingen minipig: results from a pilot study. Ann N Y Acad Sci. 2002;967:414–23. Epub 2002/06/25. doi: 10.1111/j.1749-6632.2002.tb04297.x . 12079869

[16] Renner S, Blutke A, Dobenecker B, Dhom G, Müller TD, Finan B, et al. Metabolic syndrome and extensive adipose tissue inflammation in morbidly obese Göttingen minipigs. Mol Metab. 2018;16:180–90. Epub 2018/07/19. doi: 10.1016/j.molmet.2018.06.015 ; PubMed Central PMCID: PMC6157610. 30017782 PMC6157610

[17] Rodgaard T, Skovgaard K, Moesgaard SG, Cirera S, Christoffersen BO, Heegaard PM. Extensive changes in innate immune gene expression in obese Gottingen minipigs do not lead to changes in concentrations of circulating cytokines and acute phase proteins. Anim Genet. 2014;45(1):67–73. Epub 2013/10/11. doi: 10.1111/age.12090 . 24106888

[18] Pedersen R, Ingerslev HC, Sturek M, Alloosh M, Cirera S, Christoffersen B, et al. Characterisation of gut microbiota in Ossabaw and Göttingen minipigs as models of obesity and metabolic syndrome. PLoS One. 2013;8(2):e56612. Epub 2013/02/26. doi: 10.1371/journal.pone.0056612 ; PubMed Central PMCID: PMC3577853. 23437186 PMC3577853

[19] Lützhøft DO, Sinioja T, Christoffersen B, Jakobsen RR, Geng D, Ahmad HFB, et al. Marked gut microbiota dysbiosis and increased imidazole propionate are associated with a NASH Göttingen Minipig model. BMC Microbiol. 2022;22(1):287. Epub 2022/12/02. doi: 10.1186/s12866-022-02704-w ; PubMed Central PMCID: PMC9717514. 36456963 PMC9717514

[20] Curtasu MV, Tafintseva V, Bendiks ZA, Marco ML, Kohler A, Xu Y, et al. Obesity-Related Metabolome and Gut Microbiota Profiles of Juvenile Göttingen Minipigs-Long-Term Intake of Fructose and Resistant Starch. Metabolites. 2020;10(11). Epub 2020/11/18. doi: 10.3390/metabo10110456 ; PubMed Central PMCID: PMC7697781. 33198236 PMC7697781

[21] Christoffersen BO, Gade LP, Golozoubova V, Svendsen O, Raun K. Influence of castration-induced testosterone and estradiol deficiency on obesity and glucose metabolism in male Göttingen minipigs. Steroids. 2010;75(10):676–84. Epub 2010/04/28. doi: 10.1016/j.steroids.2010.04.004 . 20420845

[22] Christoffersen B, Skyggebjerg RB, Bugge A, Kirk RK, Vestergaard B, Uldam HK, et al. Long-acting CCK analogue NN9056 lowers food intake and body weight in obese Göttingen Minipigs. Int J Obes (Lond). 2020;44(2):447–56. Epub 2019/06/09. doi: 10.1038/s41366-019-0386-0 ; PubMed Central PMCID: PMC6997118. 31175319 PMC6997118

[23] Skallerup P, Nejsum P, Cirera S, Skovgaard K, Pipper CB, Fredholm M, et al. Transcriptional immune response in mesenteric lymph nodes in pigs with different levels of resistance to Ascaris suum. Acta Parasitol. 2017;62(1):141–53. Epub 2016/12/29. doi: 10.1515/ap-2017-0017 . 28030356

[24] Kristensen T, Fredholm M, Cirera S. Expression study of GLUT4 translocation-related genes in a porcine pre-diabetic model. Mamm Genome. 2015;26(11–12):650–7. Epub 2015/09/09. doi: 10.1007/s00335-015-9601-z . 26346769

[25] Krych Ł, Kot W, Bendtsen KMB, Hansen AK, Vogensen FK, Nielsen DS. Have you tried spermine? A rapid and cost-effective method to eliminate dextran sodium sulfate inhibition of PCR and RT-PCR. J Microbiol Methods. 2018;144:1–7. Epub 2017/11/07. doi: 10.1016/j.mimet.2017.10.015 . 29107603

[26] Paulson JN PMaBH. metagenomeSeq: Statistical analysis for sparse high-throughput sequncing: http://www.cbcb.umd.edu/software/metagenomeSeq; 2013.

[27] Pedersen HD, Galsgaard ED, Christoffersen B, Cirera S, Holst D, Fredholm M, et al. NASH-inducing Diets in Göttingen Minipigs. J Clin Exp Hepatol. 2020;10(3):211–21. Epub 2020/05/15. doi: 10.1016/j.jceh.2019.09.004 ; PubMed Central PMCID: PMC7212300. 32405177 PMC7212300

[28] Koh A, Molinaro A, Ståhlman M, Khan MT, Schmidt C, Mannerås-Holm L, et al. Microbially Produced Imidazole Propionate Impairs Insulin Signaling through mTORC1. Cell. 2018;175(4):947–61.e17. Epub 2018/11/08. doi: 10.1016/j.cell.2018.09.055 . 30401435

[29] Denti P, Bertoldo A, Vicini P, Cobelli C. Identification of IVGTT minimal glucose model by nonlinear mixed-effects approaches. Conf Proc IEEE Eng Med Biol Soc. 2006;2006:5049–52. Epub 2007/10/20. doi: 10.1109/IEMBS.2006.259555 . 17947129

[30] Bergman RN, Phillips LS, Cobelli C. Physiologic evaluation of factors controlling glucose tolerance in man: measurement of insulin sensitivity and beta-cell glucose sensitivity from the response to intravenous glucose. J Clin Invest. 1981;68(6):1456–67. Epub 1981/12/01. doi: 10.1172/jci110398 ; PubMed Central PMCID: PMC370948. 7033284 PMC370948

[31] Rodríguez-López JM, Lachica M, González-Valero L, Fernández-Fígares I. Determining insulin sensitivity from glucose tolerance tests in Iberian and landrace pigs. PeerJ. 2021;9:e11014. Epub 2021/04/16. doi: 10.7717/peerj.11014 ; PubMed Central PMCID: PMC7955676. 33854837 PMC7955676

[32] Matsuda M, DeFronzo RA. Insulin sensitivity indices obtained from oral glucose tolerance testing: comparison with the euglycemic insulin clamp. Diabetes Care. 1999;22(9):1462–70. Epub 1999/09/10. doi: 10.2337/diacare.22.9.1462 . 10480510

[33] Segata N, Izard J, Waldron L, Gevers D, Miropolsky L, Garrett WS, et al. Metagenomic biomarker discovery and explanation. Genome Biol. 2011;12(6):R60. Epub 2011/06/28. doi: 10.1186/gb-2011-12-6-r60 ; PubMed Central PMCID: PMC3218848. 21702898 PMC3218848

[34] Benjamini Y, Hochberg Y. Controlling the False Discovery Rate: A Practical and Powerful Approach to Multiple Testing. Journal of the Royal Statistical Society: Series B (Methodological). 1995;57(1):289–300. doi: 10.1111/j.2517-6161.1995.tb02031.x

[35] Larsen MO, Rolin B. Use of the Göttingen Minipig as a Model of Diabetes, with Special Focus on Type 1 Diabetes Research. ILAR Journal. 2004;45(3):303–13. doi: 10.1093/ilar.45.3.303 15229377

[36] Viuff BM, Straarup EM, Nowak J, Morgills L, Skydsgaard M, Sjögren I, et al. Lipid Embolism in Obese Göttingen Minipigs. Toxicol Pathol. 2020;48(2):379–92. Epub 2019/10/28. doi: 10.1177/0192623319880464 . 31645215

[37] Christoffersen B, Golozoubova V, Pacini G, Svendsen O, Raun K. The young Göttingen minipig as a model of childhood and adolescent obesity: influence of diet and gender. Obesity (Silver Spring). 2013;21(1):149–58. Epub 2013/03/19. doi: 10.1002/oby.20249 . 23505180

[38] Mármol-Sánchez E, Artman JS, Fredholm M, Cirera S. Unraveling molecular mechanisms involved in the development of leptin resistance using the pig as a model. Anim Genet. 2021;52(1):55–65. Epub 2020/12/17. doi: 10.1111/age.13028 . 33325551

[39] Hamilton BS, Paglia D, Kwan AYM, Deitel M. Increased obese mRNA expression in omental fat cells from massively obese humans. Nature Medicine. 1995;1(9):953–6. doi: 10.1038/nm0995-953 7585224

[40] Cirera S, Jensen MS, Elbrønd VS, Moesgaard SG, Christoffersen B, Kadarmideen HN, et al. Expression studies of six human obesity-related genes in seven tissues from divergent pig breeds. Anim Genet. 2014;45(1):59–66. Epub 2013/09/17. doi: 10.1111/age.12082 . 24033492

[41] Myers MG Jr., Leibel RL, Seeley RJ, Schwartz MW. Obesity and leptin resistance: distinguishing cause from effect. Trends Endocrinol Metab. 2010;21(11):643–51. Epub 2010/09/18. doi: 10.1016/j.tem.2010.08.002 ; PubMed Central PMCID: PMC2967652. 20846876 PMC2967652

[42] Ovilo C, Fernández A, Noguera JL, Barragán C, Letón R, Rodríguez C, et al. Fine mapping of porcine chromosome 6 QTL and LEPR effects on body composition in multiple generations of an Iberian by Landrace intercross. Genet Res. 2005;85(1):57–67. Epub 2005/08/11. doi: 10.1017/s0016672305007330 . 16089036

[43] Liu M, Bai J, He S, Villarreal R, Hu D, Zhang C, et al. Grb10 promotes lipolysis and thermogenesis by phosphorylation-dependent feedback inhibition of mTORC1. Cell Metab. 2014;19(6):967–80. Epub 2014/04/22. doi: 10.1016/j.cmet.2014.03.018 ; PubMed Central PMCID: PMC4064112. 24746805 PMC4064112

[44] Nagashima S, Yagyu H, Tozawa R, Tazoe F, Takahashi M, Kitamine T, et al. Plasma cholesterol-lowering and transient liver dysfunction in mice lacking squalene synthase in the liver. J Lipid Res. 2015;56(5):998–1005. Epub 2015/03/11. doi: 10.1194/jlr.M057406 ; PubMed Central PMCID: PMC4409289. 25755092 PMC4409289

[45] Ding J, Reynolds LM, Zeller T, Müller C, Lohman K, Nicklas BJ, et al. Alterations of a Cellular Cholesterol Metabolism Network Are a Molecular Feature of Obesity-Related Type 2 Diabetes and Cardiovascular Disease. Diabetes. 2015;64(10):3464–74. Epub 2015/07/15. doi: 10.2337/db14-1314 ; PubMed Central PMCID: PMC4587646. 26153245 PMC4587646

[46] Cirera S, Taşöz E, Juul Jacobsen M, Schumacher-Petersen C, Østergaard Christoffersen B, Kaae Kirk R, et al. The expression signatures in liver and adipose tissue from obese Göttingen Minipigs reveal a predisposition for healthy fat accumulation. Nutr Diabetes. 2020;10(1):9. Epub 2020/03/25. doi: 10.1038/s41387-020-0112-y ; PubMed Central PMCID: PMC7090036. 32205840 PMC7090036

[47] Karlsson FH, Tremaroli V, Nookaew I, Bergström G, Behre CJ, Fagerberg B, et al. Gut metagenome in European women with normal, impaired and diabetic glucose control. Nature. 2013;498(7452):99–103. Epub 2013/05/31. doi: 10.1038/nature12198 . 23719380

[48] Pedersen HK, Gudmundsdottir V, Nielsen HB, Hyotylainen T, Nielsen T, Jensen BA, et al. Human gut microbes impact host serum metabolome and insulin sensitivity. Nature. 2016;535(7612):376–81. Epub 2016/07/15. doi: 10.1038/nature18646 . 27409811

[49] Sokolowska E, Blachnio-Zabielska A. The Role of Ceramides in Insulin Resistance. Front Endocrinol (Lausanne). 2019;10:577. Epub 2019/09/10. doi: 10.3389/fendo.2019.00577 ; PubMed Central PMCID: PMC6712072. 31496996 PMC6712072

[50] Pietiläinen KH, Sysi-Aho M, Rissanen A, Seppänen-Laakso T, Yki-Järvinen H, Kaprio J, et al. Acquired obesity is associated with changes in the serum lipidomic profile independent of genetic effects—a monozygotic twin study. PLoS One. 2007;2(2):e218. Epub 2007/02/15. doi: 10.1371/journal.pone.0000218 ; PubMed Central PMCID: PMC1789242. PMC1789242 17299598

[51] Petkevicius K, Virtue S, Bidault G, Jenkins B, Çubuk C, Morgantini C, et al. Accelerated phosphatidylcholine turnover in macrophages promotes adipose tissue inflammation in obesity. Elife. 2019;8. Epub 2019/08/17. doi: 10.7554/eLife.47990 ; PubMed Central PMCID: PMC6748830. 31418690 PMC6748830

[52] Tonks KT, Coster AC, Christopher MJ, Chaudhuri R, Xu A, Gagnon-Bartsch J, et al. Skeletal muscle and plasma lipidomic signatures of insulin resistance and overweight/obesity in humans. Obesity (Silver Spring). 2016;24(4):908–16. Epub 2016/02/27. doi: 10.1002/oby.21448 ; PubMed Central PMCID: PMC6585800. 26916476 PMC6585800

[53] Ader M, Pacini G, Yang YJ, Bergman RN. Importance of glucose per se to intravenous glucose tolerance. Comparison of the minimal-model prediction with direct measurements. Diabetes. 1985;34(11):1092–103. Epub 1985/11/01. doi: 10.2337/diab.34.11.1092 . 2864297

[54] Morettini M, Di Nardo F, Ingrillini L, Fioretti S, Göbl C, Kautzky-Willer A, et al. Glucose effectiveness and its components in relation to body mass index. Eur J Clin Invest. 2019;49(6):e13099. Epub 2019/03/07. doi: 10.1111/eci.13099 . 30838644

[55] Nagasaka S, Tokuyama K, Kusaka I, Hayashi H, Rokkaku K, Nakamura T, et al. Endogenous glucose production and glucose effectiveness in type 2 diabetic subjects derived from stable-labeled minimal model approach. Diabetes. 1999;48(5):1054–60. doi: 10.2337/diabetes.48.5.1054 10331410

[56] Williams JW, Zimmet PZ, Shaw JE, de Courten MP, Cameron AJ, Chitson P, et al. Gender differences in the prevalence of impaired fasting glycaemia and impaired glucose tolerance in Mauritius. Does sex matter? Diabet Med. 2003;20(11):915–20. Epub 2003/11/25. doi: 10.1046/j.1464-5491.2003.01059.x . 14632717

[57] De Pergola G, Di Roma P, Paoli G, Guida P, Pannacciulli N, Giorgino R. Haptoglobin serum levels are independently associated with insulinemia in overweight and obese women. J Endocrinol Invest. 2007;30(5):399–403. Epub 2007/06/30. doi: 10.1007/BF03346317 . 17598972

[58] Færch K, Vistisen D, Pacini G, Torekov SS, Johansen NB, Witte DR, et al. Insulin Resistance Is Accompanied by Increased Fasting Glucagon and Delayed Glucagon Suppression in Individuals With Normal and Impaired Glucose Regulation. Diabetes. 2016;65(11):3473–81. Epub 2016/08/10. doi: 10.2337/db16-0240 . 27504013

[59] Yoo S, Kim D, Koh G. The Change in Glucagon Following Meal Ingestion Is Associated with Glycemic Control, but Not with Incretin, in People with Diabetes. J Clin Med. 2021;10(11). Epub 2021/07/03. doi: 10.3390/jcm10112487 ; PubMed Central PMCID: PMC8200068. 34199839 PMC8200068

[60] Honzawa N, Fujimoto K, Kitamura T. Cell Autonomous Dysfunction and Insulin Resistance in Pancreatic α Cells. Int J Mol Sci. 2019;20(15). Epub 2019/07/31. doi: 10.3390/ijms20153699 ; PubMed Central PMCID: PMC6695724. 31357734 PMC6695724

[61] Adeva-Andany MM, Funcasta-Calderón R, Fernández-Fernández C, Castro-Quintela E, Carneiro-Freire N. Metabolic effects of glucagon in humans. J Clin Transl Endocrinol. 2019;15:45–53. Epub 2019/01/09. doi: 10.1016/j.jcte.2018.12.005 ; PubMed Central PMCID: PMC6312800. 30619718 PMC6312800

[62] Clarke SF, Murphy EF, Nilaweera K, Ross PR, Shanahan F, O’Toole PW, et al. The gut microbiota and its relationship to diet and obesity: new insights. Gut Microbes. 2012;3(3):186–202. Epub 2012/05/11. doi: 10.4161/gmic.20168 ; PubMed Central PMCID: PMC3427212. 22572830 PMC3427212

[63] Crovesy L, Masterson D, Rosado EL. Profile of the gut microbiota of adults with obesity: a systematic review. Eur J Clin Nutr. 2020;74(9):1251–62. Epub 2020/04/02. doi: 10.1038/s41430-020-0607-6 . 32231226

[64] Duan M, Wang Y, Zhang Q, Zou R, Guo M, Zheng H. Characteristics of gut microbiota in people with obesity. PLoS One. 2021;16(8):e0255446. Epub 2021/08/11. doi: 10.1371/journal.pone.0255446 ; PubMed Central PMCID: PMC8354443. 34375351 PMC8354443

[65] Yassour M, Lim MY, Yun HS, Tickle TL, Sung J, Song YM, et al. Sub-clinical detection of gut microbial biomarkers of obesity and type 2 diabetes. Genome Med. 2016;8(1):17. Epub 2016/02/18. doi: 10.1186/s13073-016-0271-6 ; PubMed Central PMCID: PMC4756455. 26884067 PMC4756455

[66] Zhang X, Shen D, Fang Z, Jie Z, Qiu X, Zhang C, et al. Human gut microbiota changes reveal the progression of glucose intolerance. PLoS One. 2013;8(8):e71108. Epub 2013/09/10. doi: 10.1371/journal.pone.0071108 ; PubMed Central PMCID: PMC3754967. 24013136 PMC3754967

[67] Qin J, Li Y, Cai Z, Li S, Zhu J, Zhang F, et al. A metagenome-wide association study of gut microbiota in type 2 diabetes. Nature. 2012;490(7418):55–60. Epub 2012/10/02. doi: 10.1038/nature11450 . 23023125

[68] Ngowi EE, Wang YZ, Khattak S, Khan NH, Mahmoud SSM, Helmy Y, et al. Impact of the factors shaping gut microbiota on obesity. J Appl Microbiol. 2021;131(5):2131–47. Epub 2021/02/12. doi: 10.1111/jam.15036 . 33570819

[69] Nirmalkar K, Murugesan S, Pizano-Zárate ML, Villalobos-Flores LE, García-González C, Morales-Hernández RM, et al. Gut Microbiota and Endothelial Dysfunction Markers in Obese Mexican Children and Adolescents. Nutrients. 2018;10(12). Epub 2018/12/24. doi: 10.3390/nu10122009 ; PubMed Central PMCID: PMC6315777. 30572569 PMC6315777

[70] Foley KP, Zlitni S, Duggan BM, Barra NG, Anhê FF, Cavallari JF, et al. Gut microbiota impairs insulin clearance in obese mice. Mol Metab. 2020;42:101067. Epub 2020/08/30. doi: 10.1016/j.molmet.2020.101067 ; PubMed Central PMCID: PMC7522491. 32860984 PMC7522491

[71] Golloso-Gubat MJ, Ducarmon QR, Tan RCA, Zwittink RD, Kuijper EJ, Nacis JS, et al. Gut Microbiota and Dietary Intake of Normal-Weight and Overweight Filipino Children. Microorganisms. 2020;8(7). Epub 2020/07/12. doi: 10.3390/microorganisms8071015 ; PubMed Central PMCID: PMC7409305. 32650516 PMC7409305

[72] Pinart M, Dötsch A, Schlicht K, Laudes M, Bouwman J, Forslund SK, et al. Gut Microbiome Composition in Obese and Non-Obese Persons: A Systematic Review and Meta-Analysis. Nutrients. 2021;14(1). Epub 2022/01/12. doi: 10.3390/nu14010012 ; PubMed Central PMCID: PMC8746372. 35010887 PMC8746372

[73] Ozato N, Saito S, Yamaguchi T, Katashima M, Tokuda I, Sawada K, et al. Blautia genus associated with visceral fat accumulation in adults 20–76 years of age. npj Biofilms and Microbiomes. 2019;5(1):28. doi: 10.1038/s41522-019-0101-x 31602309 PMC6778088

[74] Hosomi K, Saito M, Park J, Murakami H, Shibata N, Ando M, et al. Oral administration of Blautia wexlerae ameliorates obesity and type 2 diabetes via metabolic remodeling of the gut microbiota. Nat Commun. 2022;13(1):4477. Epub 2022/08/19. doi: 10.1038/s41467-022-32015-7 ; PubMed Central PMCID: PMC9388534. 35982037 PMC9388534

[75] Agus A, Clément K, Sokol H. Gut microbiota-derived metabolites as central regulators in metabolic disorders. Gut. 2021;70(6):1174–82. Epub 2020/12/05. doi: 10.1136/gutjnl-2020-323071 ; PubMed Central PMCID: PMC8108286. 33272977 PMC8108286

[76] Pallister T, Jackson MA, Martin TC, Zierer J, Jennings A, Mohney RP, et al. Hippurate as a metabolomic marker of gut microbiome diversity: Modulation by diet and relationship to metabolic syndrome. Sci Rep. 2017;7(1):13670. Epub 2017/10/24. doi: 10.1038/s41598-017-13722-4 . 29057986 PMC5651863

[77] Dumas ME, Rothwell AR, Hoyles L, Aranias T, Chilloux J, Calderari S, et al. Microbial-Host Co-metabolites Are Prodromal Markers Predicting Phenotypic Heterogeneity in Behavior, Obesity, and Impaired Glucose Tolerance. Cell Rep. 2017;20(1):136–48. Epub 2017/07/07. doi: 10.1016/j.celrep.2017.06.039 ; PubMed Central PMCID: PMC5507771. 28683308 PMC5507771

[78] Krueger ES, Lloyd TS, Tessem JS. The Accumulation and Molecular Effects of Trimethylamine N-Oxide on Metabolic Tissues: It’s Not All Bad. Nutrients. 2021;13(8). Epub 2021/08/28. doi: 10.3390/nu13082873 ; PubMed Central PMCID: PMC8400152. 34445033 PMC8400152

[79] Wang F, Baden MY, Guasch-Ferré M, Wittenbecher C, Li J, Li Y, et al. Plasma metabolite profiles related to plant-based diets and the risk of type 2 diabetes. Diabetologia. 2022;65(7):1119–32. doi: 10.1007/s00125-022-05692-8 35391539 PMC9810389

[80] Zhou J, Zhou S, Zeng S. Experimental diabetes treated with trigonelline: effect on β cell and pancreatic oxidative parameters. Fundam Clin Pharmacol. 2013;27(3):279–87. Epub 2011/12/17. doi: 10.1111/j.1472-8206.2011.01022.x . 22172053

[81] Calvani R, Miccheli A, Capuani G, Tomassini Miccheli A, Puccetti C, Delfini M, et al. Gut microbiome-derived metabolites characterize a peculiar obese urinary metabotype. Int J Obes (Lond). 2010;34(6):1095–8. Epub 2010/03/10. doi: 10.1038/ijo.2010.44 . 20212498

[82] Furuhashi M, Matsumoto M, Tanaka M, Moniwa N, Murase T, Nakamura T, et al. Plasma Xanthine Oxidoreductase Activity as a Novel Biomarker of Metabolic Disorders in a General Population. Circ J. 2018;82(7):1892–9. Epub 2018/04/13. doi: 10.1253/circj.CJ-18-0082 . 29643318

[83] Molinaro A, Bel Lassen P, Henricsson M, Wu H, Adriouch S, Belda E, et al. Imidazole propionate is increased in diabetes and associated with dietary patterns and altered microbial ecology. Nat Commun. 2020;11(1):5881. Epub 2020/11/20. doi: 10.1038/s41467-020-19589-w ; PubMed Central PMCID: PMC7676231. 33208748 PMC7676231

[84] van Son J, Serlie MJ, Ståhlman M, Bäckhed F, Nieuwdorp M, Aron-Wisnewsky J. Plasma Imidazole Propionate Is Positively Correlated with Blood Pressure in Overweight and Obese Humans. Nutrients. 2021;13(8). Epub 2021/08/28. doi: 10.3390/nu13082706 ; PubMed Central PMCID: PMC8399073. 34444866 PMC8399073

[85] Fernández-Veledo S, Vendrell J. Gut microbiota-derived succinate: Friend or foe in human metabolic diseases? Rev Endocr Metab Disord. 2019;20(4):439–47. Epub 2019/10/28. doi: 10.1007/s11154-019-09513-z ; PubMed Central PMCID: PMC6938788. 31654259 PMC6938788

[86] Benus RF, van der Werf TS, Welling GW, Judd PA, Taylor MA, Harmsen HJ, et al. Association between Faecalibacterium prausnitzii and dietary fibre in colonic fermentation in healthy human subjects. Br J Nutr. 2010;104(5):693–700. Epub 2010/03/30. doi: 10.1017/S0007114510001030 . 20346190

[87] Varel VH, Yen JT. Microbial perspective on fiber utilization by swine. J Anim Sci. 1997;75(10):2715–22. Epub 1997/10/23. doi: 10.2527/1997.75102715x . 9331875

[88] Fu J, Zheng Y, Gao Y, Xu W. Dietary Fiber Intake and Gut Microbiota in Human Health. Microorganisms. 2022;10(12). Epub 2022/12/24. doi: 10.3390/microorganisms10122507 ; PubMed Central PMCID: PMC9787832. 36557760 PMC9787832

[89] Frost G, Sleeth ML, Sahuri-Arisoylu M, Lizarbe B, Cerdan S, Brody L, et al. The short-chain fatty acid acetate reduces appetite via a central homeostatic mechanism. Nature Communications. 2014;5(1):3611. doi: 10.1038/ncomms4611 24781306 PMC4015327

[90] Thakare R, Alamoudi JA, Gautam N, Rodrigues AD, Alnouti Y. Species differences in bile acids I. Plasma and urine bile acid composition. J Appl Toxicol. 2018;38(10):1323–35. Epub 2018/05/23. doi: 10.1002/jat.3644 . 29785833

[91] Spinelli V, Lalloyer F, Baud G, Osto E, Kouach M, Daoudi M, et al. Influence of Roux-en-Y gastric bypass on plasma bile acid profiles: a comparative study between rats, pigs and humans. Int J Obes (Lond). 2016;40(8):1260–7. Epub 2016/04/20. doi: 10.1038/ijo.2016.46 . 27089995

