# Supplementary material for: High fat diet is associated with gut microbiota dysbiosis and decreased gut microbial derived metabolites related to metabolic health in young Göttingen Minipigs
Source: PLoS One. 2024 Mar 1;19(3):e0298602. doi: 10.1371/journal.pone.0298602 (PMC10906878; doi:10.1371/journal.pone.0298602)
Supplement: S1 File — (PDF) [file pone.0298602.s001.pdf]

Supplementary Methods

Zoletil mixture used for anaesthesia during castration/ovariectomy, DEXA scanning, catheter placement and in relation to euthanasia:

Zoletil mixture was used to induce general anaesthesia and analgesia. The mixture contained 125 mg zolazepam and 125 mg tiletamine (dry powder from Zoletil 50 vet., ChemVet, Denmark), ketamine (1.25 mL Ketaminol Vet. 100 mg/L, Intervet, Denmark) xylacin (6.5 mL Rompun Vet, 20 mg/mL) Bayer A/S, Denmark) and buthorphanol (2.5 mL Torbugesic, 10 mg/mL, ScanVet, Denmark). The mixture was given intra-muscularly (i.m.) in a dose of 1 ml/10-15 kg body weight (BW).

Animal study procedures for castration and ovariectomized

Twelve young male and 12 young female Göttingen Minipigs (Ellegaard Göttingen Minipigs A/S, Dalmose, Denmark). The health status of the pigs can be found on Ellegaard Göttingen Minipigs home page: https://minipigs.dk/about-gottingen-minipigs/health-status. The pigs were either castrated or ovariectomized (OVX) 11 days prior to study start at which point they were 9 weeks of age. Before surgery the pigs were acclimated for 9 days to the facility.
Anaesthesia was induced with Zoletil-mixture for minipigs in a dose of 0.1 ml/kg given i.m.
After the pig was anaesthetised an injection of 1 mg atropine/mL (0.02 mL/kg) was given i.m. For ovariectomy, the pig was hereafter intubated, and the anaesthesia was maintained on a mixture of isoflurane and oxygen. For castration, the procedure was quick and performed under the Zoletil mix anaesthesia.

Amoxicillin (Curamox Prolongatum Vet, 150 mg/ml) was given i.m. in a dose of 0.1ml/kg before start of surgery and again 48 h later. As analgesia Meloxicam (Metacam® inj., 20 mg/ml, 0.4 mg/kg i.m.) was given preoperatively, followed Metacam oral suspension (15 mg/ml, 0.03 ml/kg) given for 3 days post-operatively.

The surgical region was shaved, washed thoroughly with soap and water and disinfected with 70 % ethanol or iodine, where after sterile drapes were used to secure aseptic access to the entire surgery field.

For ovariectomy, the ovaries were localised and isolated by placing a haemostatic forceps on the salpinx and ovarian artery without damaging the uterine horn. Then the ovaries were cut off using ultrasonic shears (Harmonic ACE®). After inspection to ensure proper haemostasis and removal of all ovarian tissue, the procedure was repeated on the second ovary. Lastly, the abdominal incision was closed in 2-3 layers. For castration, a scrotal incision was made over the testicle through the skin, subcutaneous tissue and tunica vaginalis to expose the testicle. The spermatic cord was cut by ultrasonic shears (Harmonic ACE®) assuring proper haemostasis. After repeating the procedure on the second testicle, the incision was closed in 2-3 layers.

Implantation of the central-venous catheter

Central venous catheters were implanted through an ear vein after approx. 11 weeks of diet feeding (Day 78-80). During the catheter implantation, the pigs were anaesthetized i.m. with 1 mL/10 kg of the Zoletil mixture described above. In addition, the pigs were given an i.m. injection of 1 mg atropine/mL (0.01 mL/kg). A central venous catheter (BD Careflow 3Fr 200mm, Argon Medical, Texas, USA) was implanted in v. jugularis via an ear vein using a minimally invasive technique. The catheter was fixed using a special plate and steel piercings. After implantation of

the catheter and the piercings and on the following two days, the pigs were treated i.m. with 0.4 mg/kg meloxicam (Metacam®, 20 mg/ml). The catheter was used for stress-free blood sampling during the metabolic tests.

Mixed meal test (MMT)

The mixed meal was prepared as follows: The same amount of each of the following ingredients were mixed: Skyr (Løgismose, 10 g protein/100 g), double cream (38 g fat/100 g) and powdered sugar (100 g sugar/100 g). The pigs were fed 6 g/kg of the mixture, resulting in the following doses of macronutrients: 0.24 g protein/kg, 0.76 g fat/kg and 2.1 g glucose/kg and the clock was started when the meal was finished. Blood (2 mL) was sampled at the following time points in relation to the meal: Pre-dose and 15, 30, 45, 60, 90, 120, 150 and 180 minutes. The blood samples were transferred to EDTA tubes with 25 µl special stabilization buffer/ml blood (50 ul in total for the 2 ml blood) (see below for description if the stabilization buffer).

Stabilization buffer

The stabilization buffer used during the MMT was prepared as follows: 6.50 g K_3EDTA (MW 406.53), Fluka 03665, is dissolved in 50 mL Aprotinin, 10.000 KIU/mL, and 0.5 mL 40 mM valine-pyrrolidine solution (16.48 mg Val-pyr/2 mL sterile H_2O) was added. The pH was regulated to 7.4 by 1M HCl.

Gene expression analysis

RNA was extracted from ~30 mg of liver using the RNeasy Mini Kit (Qiagen), from 100 mg of omental fat tissue using the protocol of (1) and from 100 mg skeletal muscle tissue using the RNeasy Fibrous Tissue Mini Kit (Qiagen). The GentleMACS^{TM} Octo Dissociator machine (Milteny Biotec)

with M tubes was used for homogenization. All samples were DNAse treated according to each protocol. 100 ng of each RNA sample were used to make 2 cDNA replicates using ImProm II reverse transcriptase (Promega, Denmark) and a 3:1 mixture of random hexamers/OligodT primers, according to the manufacturer's instructions. cDNA from liver was diluted 16 times, while cDNA from omentum and muscle were diluted 8 times before using in quantitative real-time PCR (qPCR). Quantity and quality of each RNA sample were assessed by NanoDrop^{TM} spectrophotometer machine (Thermo Scientific) and by the RNA 6000 Nano Kit (Agilent Technologies) in an Agilent RNA Bioanalyzer system (Agilent Technologies) respectively. Only RQI values of >6 were considered acceptable (the average values for each tissue were as follows: liver - 8.43 ± 0.23; omental fat tissue - 7.72 ± 0.66 (one pig from the female chow group was excluded due to a low RIN value); skeletal muscle tissue - 9.07 ± 0.11). Subsequently, cDNA was synthesized from 100ng of total RNA using ImPromII reverse transcriptase (Promega), RNAsin (Promega) and oligodT/random hexamers (1:3) primers following manufacturer's recommendations. Two cDNA replicates were made from each RNA sample to account for technical variation. A negative control (-RT; without reverse transcriptase) was made for each tissue to test the presence of genomic DNA. cDNA from liver was diluted 16 times, while cDNA from omentum and skeletal muscle was diluted 8 times before using in quantitative real-time PCR (qPCR).

A panel of 96 different obesity relevant genes including possible reference genes were chosen from an *in-house* primer library (Suppl. Table S2) for profiling by qPCR using the high-throughput qPCR Biomark HD platform (Fluidigm, San Francisco, California) following manufacturer's protocols.

The qPCR was performed using Flex Six IFC chips for assay optimization and 96.96 IFC chips in a Biomark^{TM} HD machine (Fluidigm) using SsoFast^{TM} EvaGreen$^{®}$ following manufacturer instructions for reactions and thermal profile.

Additionally, 7 extra genes of interest (Suppl. Table S2) were profiled at later stage solely in the liver samples using QuantiFast SYBR® Green PCR Kit (Qiagen, Germany) in a Mx3005P platform as previously described (2).

qPCR raw data was manually examined using the Fluidigm Real-Time PCR Analysis associated software or the MxPro associated software. Firstly, -RT samples and NTC were examined for no-amplification. Furthermore, samples low expressed, assays with many missing values, cDNA replicates varying more than 1.5 cycles, samples with unspecific melting curve or assays with PCR efficiencies <80% or >110%, were removed from final analysis.

Subsequently, manual curated qPCR data was processed using Genex 7.0 software (MultiID, Sweden). Briefly, Cq values from both chips were calibrated for differences in baseline correction (only for the Biomark experiment), Cq values were corrected for PCR efficiency, values were then normalized to the reference genes selected for each tissue, cDNA replicates were averaged, fold changes were calculated related to SD group and data was log2 transformed previous statistical analysis.

Biomark platform: After manual curation and processing of qPCR data, 64 assays were accepted for further analysis in liver, 56 in omentum and 51 in skeletal muscle (Suppl. Table S2) out of 96 profiled gene-assays. Beta-actin (ACTB), TATA box binding protein (TBP), & tyrosine 3-monooxygenase/tryptophan 5-monooxygenase activation protein zeta (YWHAZ), were chosen as reference genes for liver data, ACTB and YWHAZ were chosen as reference genes for omentum data and TBP and ribosomal protein L4 (RPL4) were chosen as reference genes for skeletal muscle data.

Mx3005P platform: BAAT gene was not expressed in the liver, DPBAR1 assay was not specific, and hence five genes were accepted for further analysis in liver (Suppl. Table S2). ACTB, TBP and YWHAZ were used for normalization.

A two-way ANOVA test was applied for each tissue profiled (with diet and sex as factors) with multiple test correction; but because there was not any significant difference between sexes a T-test was subsequently applied to compare chow versus HFD groups (see Suppl. Table S2).

DNA extraction, sequencing and pre-processing of raw data

Extraction of bacterial DNA was performed by using Bead-beat micro AX bacteria gravity kit (A&A biotechnology, Gdynia, Poland) according to manufacturer's instruction. DNA concentration and purity were validated using Qubit Fluorometer (Invitrogen, Carlsbad, CA, USA). Determination of gut prokaryotic composition using NexSeq (Illumina, Ca, USA) based 16S rRNA gene-amplicon sequencing by amplifying the V3 region with paired primers designed with adapters for the Nextera Index Kit® (Illumina, CA, USA): NXt_338_F: 5'- TCG TCG GCA GCG TCA GAT GTG TAT AAG AGA CAG ACW CCT ACG GGW GGC AGC AG -3' and NXt_518_R: 5'- GTC TCG TGG GCT CGG AGA TGT GTA TAA GAG ACA GAT TAC CGC GGC TGC TGG -3'. Amplification of purified DNA was done in a 25 µl final volume PCR1 reaction containing 5 µL 5xPCRBIO HiFi buffer (PCRBiosystems, PA, USA), 0.5 µL Primer mix (5µM), 0.25 µL PCRBIO HiFi Polymerase (PCRBiosystems, PA, USA), 1 µL BSA, 1 µL Formamide, 12.25 µL nuclease-free water and 5 µL of genomic DNA (1 ng/µL) run on a SureCycler 8800 (Agilent technologies, CA,USA) using the following protocol: Initial denaturation at 95 °C for 2 minutes; 33 cycles of denaturation at 95 °C for 15 seconds annealing of primer at 55 °C for 15 seconds and elongation at 72 °C for 20 seconds, ending with 72 °C for 4 minutes. Before proceeding, the integrity and concentration of PCR1 products were verified using 1.5% agarose gel and Qubit Fluorometer (Invitrogen, Carlsbad, CA, USA). PCR1 products were cleaned using Biomek 4000, Automated Laboratory Workstation (Beckman Coulter Life Science, IN, USA). For PCR2 (a final volume of 25 µL): 5 µL 5xPCRBIO HiFi buffer (PCR Biosystems, PA, USA), 4 µL of corresponding P5 and P7 (Nextera index Kit),

0.25 µL of PCRBIO HiFi Polymerase (PCR Biosystems, PA, USA), 13.75 µL of nuclease-free water and 2 µL of cleaned PCR1 product were mixed. The amplification protocol used was: 95 °C for 1 min; 13 cycles of 95 °C for 15 seconds, 55 °C for 15 seconds and 72 °C for 15 seconds; ending with 72 °C for 5 minutes using same instrument as PCR1. Subsequently an additional cleaning and concentration measurement of PCR2 products were conducted before normalization and pooling were performed using Biomek 4000, Automated Laboratory Workstation (Beckman Coulter Life Science, IN, USA). The raw dataset containing pair-ended reads was merged and trimmed using UPARSE pipeline with build-in fastq_mergepairs and fastq_filter script to generate high quality (97% similarity level) Operational Toxonomic Unit (OTU) – tables.

References.

1. Skallerup P, Nejsum P, Cirera S, Skovgaard K, Pipper CB, Fredholm M, et al. Transcriptional immune response in mesenteric lymph nodes in pigs with different levels of resistance to Ascaris suum. Acta Parasitol. 2017;62(1):141-53.

2. Kristensen T, Fredholm M, Cirera S. Expression study of GLUT4 translocation-related genes in a porcine pre-diabetic model. Mamm Genome. 2015;26(11-12):650-7.
