# Supplementary figures and images for: High fat diet is associated with gut microbiota dysbiosis and decreased gut microbial derived metabolites related to metabolic health in young Göttingen Minipigs
Source: PLoS One. 2024 Mar 1;19(3):e0298602. doi: 10.1371/journal.pone.0298602 (PMC10906878; doi:10.1371/journal.pone.0298602)

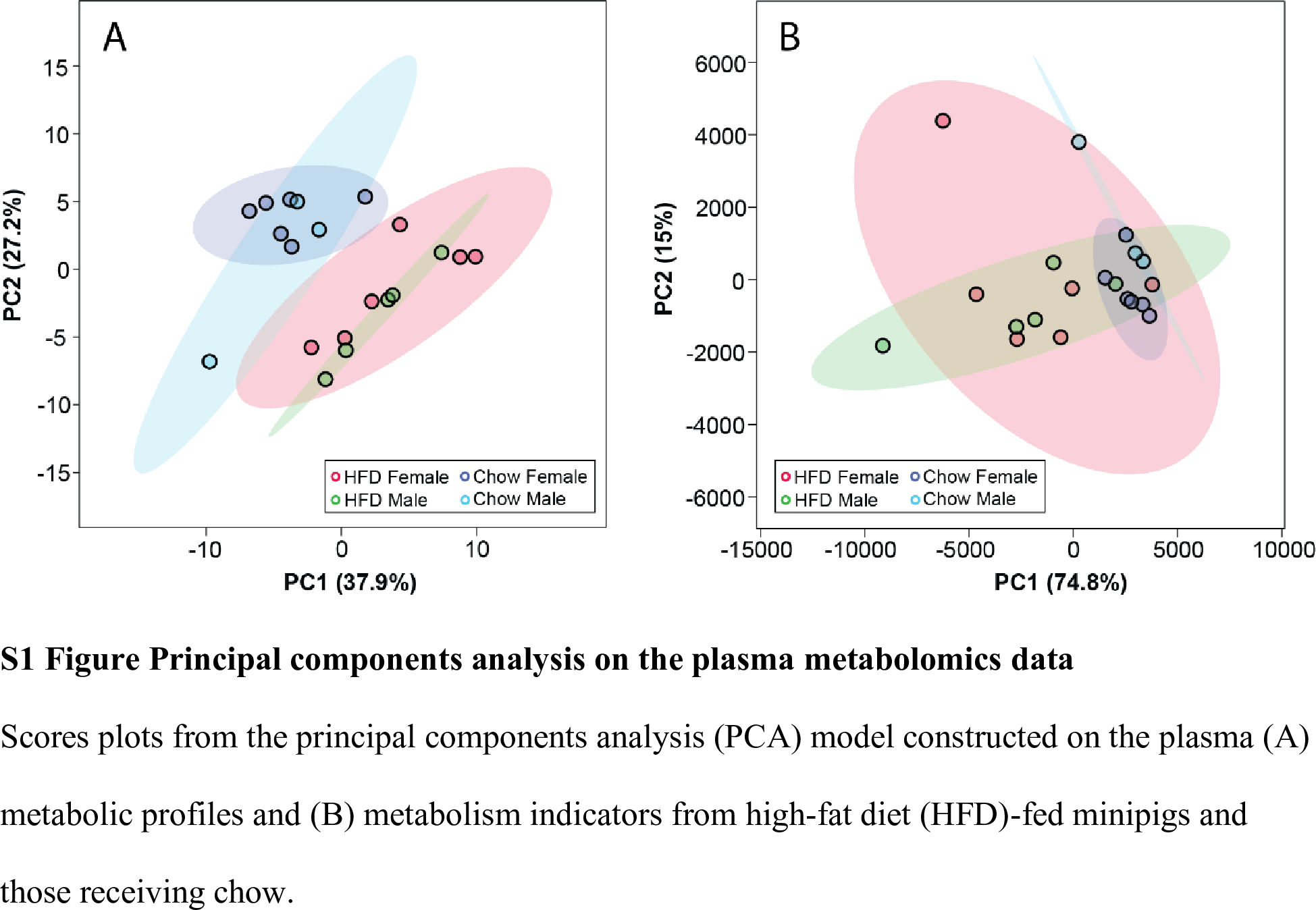

Supplement: S1 Fig — Scores plots from the principal components analysis (PCA) model constructed on the plasma (A) metabolic profiles and (B) metabolism indicators from high-fat diet (HFD)-fed minipigs and those receiving chow. (TIF) [file pone.0298602.s002.tif]

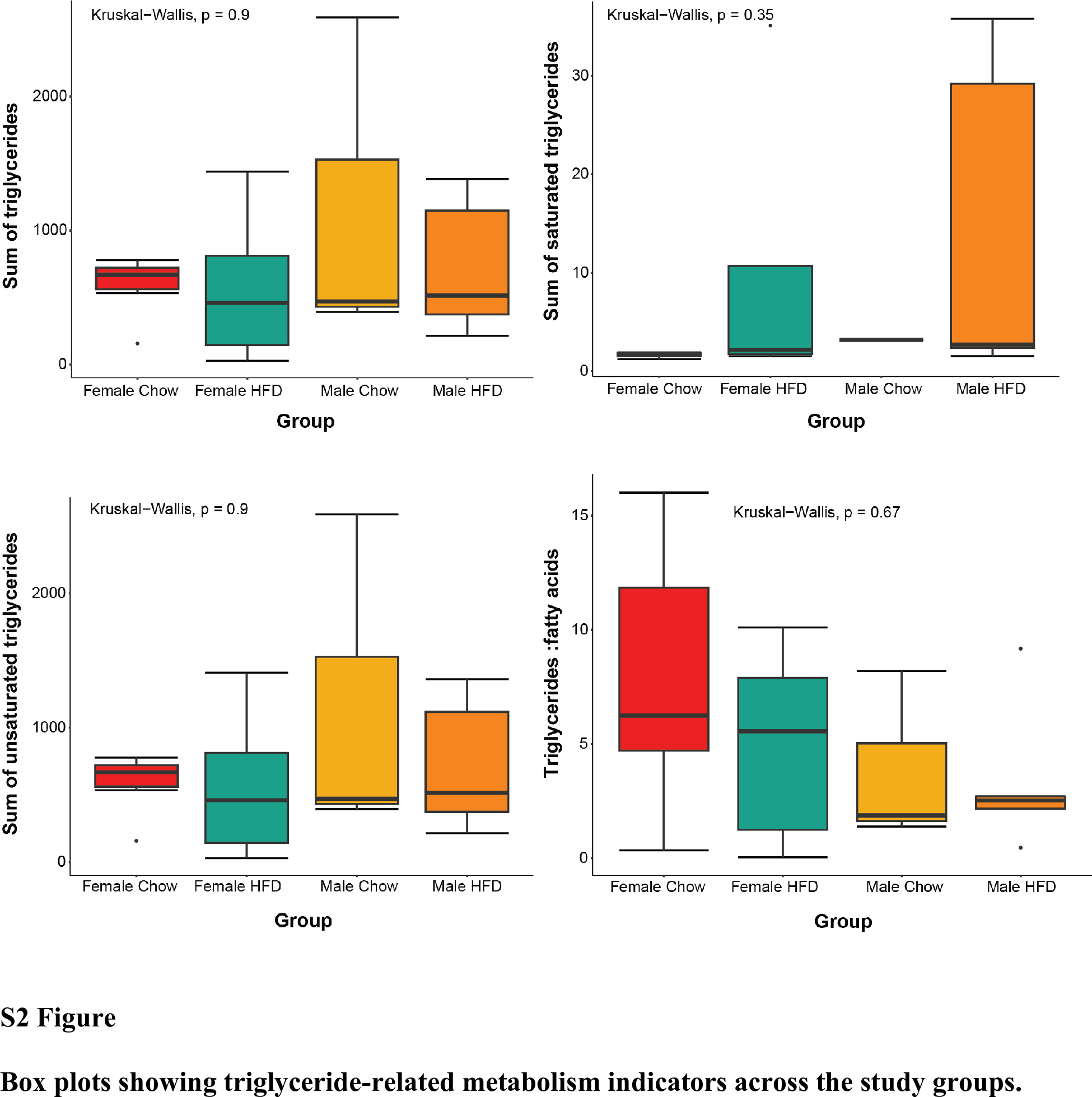

Supplement: S2 Fig — (TIF) [file pone.0298602.s003.tif]

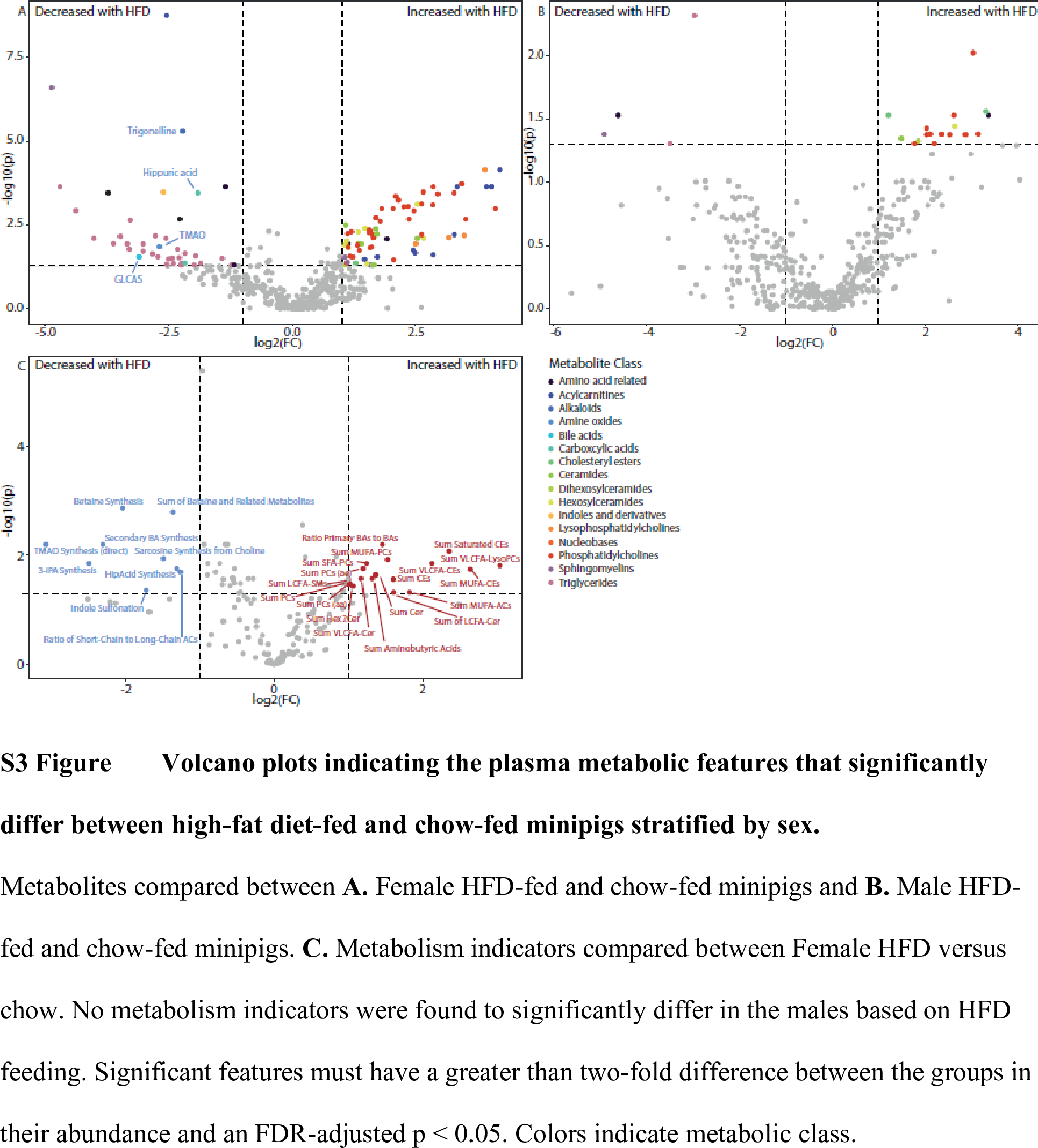

Supplement: S3 Fig — Metabolites compared between A. Female HFD-fed and chow-fed minipigs and B. Male HFD-fed and chow-fed minipigs. C. Metabolism indicators compared between Female HFD versus chow. No metabolism indicators were found to significantly differ in the males based on HFD feeding. Significant features must have a greater than two-fold difference between the groups in their abundance and an FDR-adjusted p < 0.05. Colors indicate metabolic class. (TIF) [file pone.0298602.s004.tif]

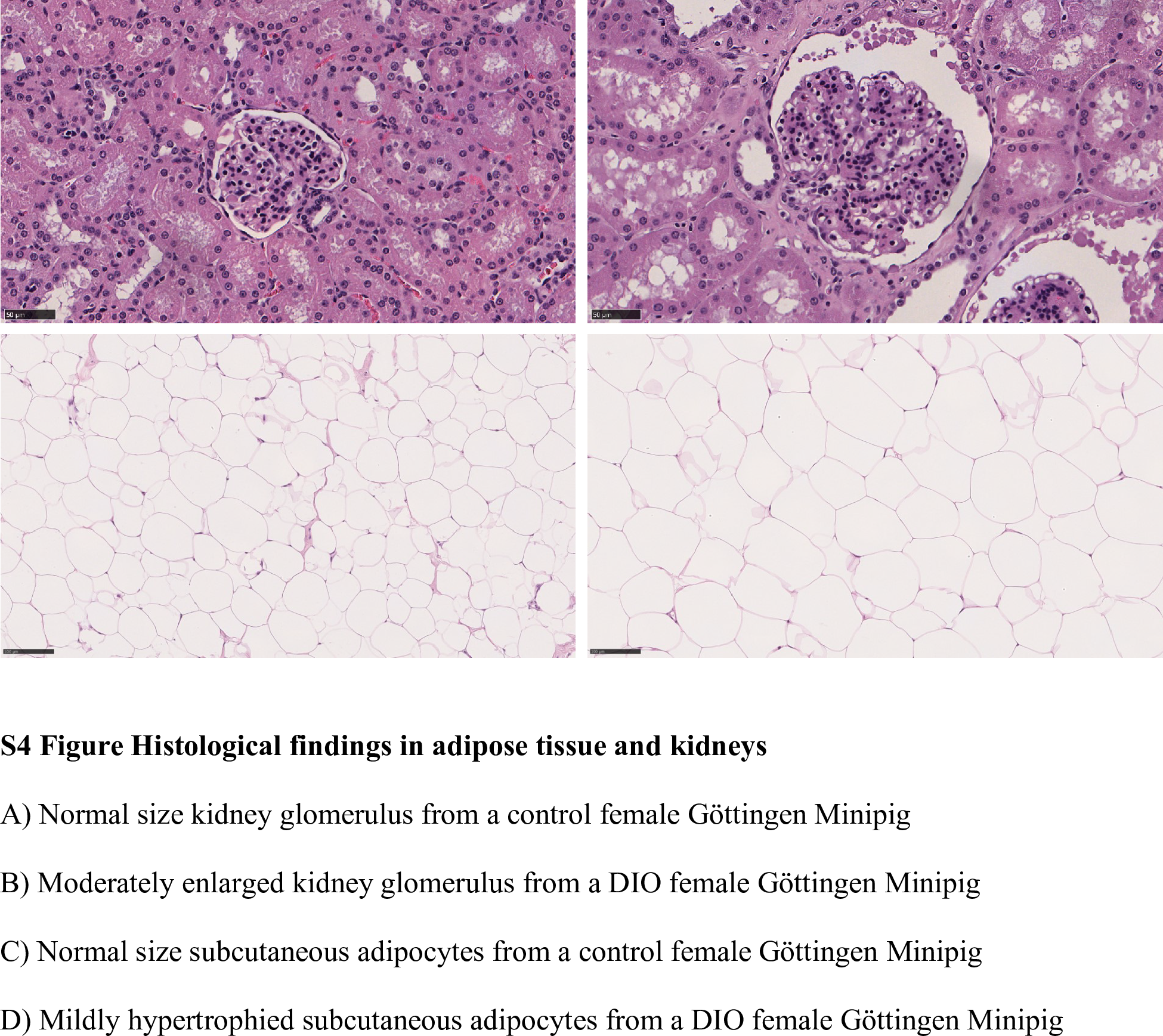

Supplement: S4 Fig — A) Normal size kidney glomerulus from a control female Göttingen Minipig, B) Moderately enlarged kidney glomerulus from a DIO female Göttingen Minipig, C) Normal size subcutaneous adipocytes from a control female Göttingen Minipig, D) Mildly hypertrophied subcutaneous adipocytes from a DIO female Göttingen Minipig. (TIF) [file pone.0298602.s005.tif]
