# Supplementary material for: High fat diet is associated with gut microbiota dysbiosis and decreased gut microbial derived metabolites related to metabolic health in young Göttingen Minipigs
Source: PLoS One. 2024 Mar 1;19(3):e0298602. doi: 10.1371/journal.pone.0298602 (PMC10906878; doi:10.1371/journal.pone.0298602)
Supplement: S1 Table — (DOCX) [file pone.0298602.s006.docx]

**S1 Table Diet specifications**

|  | **Chow^1^** | **High fat diet^2^** |
| --- | --- | --- |
| **Moisture (%)** | 10 | 6 |
| **Protein (%)** | 13 | 13 |
| **Fat (%)^3^** | 2.1 | 25 |
| **Crude Fibre (%)** | 14.5 | 2.9 |
| **Starch (%)** | 27.1 | 10.7 |
| **Added cholesterol (%)** | None | 0.5 |
| **Added Fructose (%)** | None | 23.2 |
| **Gross Energy (MJ/kg)** | 13.8 | 19.0 |

^1^ SDS minipig expanded (Special Diets Services, Scanbur, DK)

^2^ Custom made high fat diet, Foulum, Aarhus University, Aarhus, Denmark

^3^ Fat in the chow diet is primarily from sunflower and soybean, and in the high fat diet it is primarily from lard (21%) and soybean oil.

**Composition of the high fat diet:**

| **Ingredient** | **Weight percent** |
| --- | --- |
| Wheat | 5 |
| Barley | 5 |
| Oat | 12.4 |
| Fructose | 23.2 |
| Beet pulp | 6 |
| Soy oil | 3 |
| Lard | 21 |
| Feed chalk 38% | 3.1 |
| Monocalcium phosphate | 2.7 |
| Cholesterol | 0.5 |
| Minerals | 18.1 |
| **Total** | **100** |

**Composition of the chow diet:**

SDS minipig expanded (Special Diets Services, Scanbur, DK, www.sds-diets.com)
