# Supplementary material for: High fat diet is associated with gut microbiota dysbiosis and decreased gut microbial derived metabolites related to metabolic health in young Göttingen Minipigs
Source: PLoS One. 2024 Mar 1;19(3):e0298602. doi: 10.1371/journal.pone.0298602 (PMC10906878; doi:10.1371/journal.pone.0298602)
Supplement: S3 Table — (DOCX) [file pone.0298602.s008.docx]

**S3 Table Clinical chemistry and serum/plasma biomarkers.**

Castrated male (M) and ovariectomised female (F) Göttingen Minipigs fed chow or high fat diet (HFD). Mean±SD, n=5-6. Two-way ANOVA with gender and diet as explanatory variables followed by Tukeys multiple comparison test. Only the following comparisons were considered relevant: M-HFD vs. M-chow, M-HFD vs. F-HFD, M-chow vs. F-chow and F-HFD vs. F-chow. Superscript numbers indicate significant difference between groups. ^a^ p<0.05, ^aa^ p<0.01 and ^aaa^ p<0.001 for F-HFD vs. F-chow, ^b^ p<0.05, ^bb^ p<0.01 and ^bbb^ p<0.001 for M-HFD vs. M-chow.

| **Group** | **Females HFD** | | **Males HFD** | | **Females chow** | | **Males chow** | | **Two-way ANOVA p-values** | | |
| --- | --- | --- | --- | --- | --- | --- | --- | --- | --- | --- | --- |
| **Parameter** | **Mean** | **SD** | **Mean** | **SD** | **Mean** | **SD** | **Mean** | **SD** | **Diet** | **Sex** | **Sex*diet** |
| Fasting glucose (mM) | 3.8 | 0.3 | 4.0^b^ | 0.2 | 3.5 | 0.4 | 3.4 | 0.3 | <0.01 | 0.60 | 0.50 |
| Fasting insulin (pM) | 65.3^a^ | 21.0 | 42.0 | 17.8 | 27.0 | 20.0 | 21.5 | 14.4 | <0.01 | 0.08 | 0.28 |
| Fasting C-peptide (pM) | 57.2 | 18.1 | 37.2 | 13.0 | 37.4 | 20.0 | 24.4 | 8.1 | <0.05 | <0.05 | 0.81 |
| Fasting glucagon (pM) | 20.9^aa^ | 5.3 | 19.0 | 7.4 | 9.5 | 3.7 | 10.9 | 3.0 | <0.001 | 0.86 | 0.28 |
| ALT (µk/L) | 0.64^aaa^ | 0.20 | 0.74^b^ | 0.29 | 1.41 | 0.35 | 1.27 | 0.28 | <0.001 | 0.90 | 0.31 |
| AST (µk/L) | 0.33 | 0.05 | 0.36 | 0.10 | 0.42 | 0.09 | 0.40 | 0.06 | <0.05 | 0.76 | 0.55 |
| ALP (µk/L) | 1.54^aaa^ | 0.33 | 1.71 | 0.31 | 2.87 | 0.36 | 2.44 | 0.71 | <0.001 | 0.51 | 0.14 |
| GGT (µk/L) | 0.86 | 0.22 | 0.79 | 0.13 | 0.93 | 0.17 | 0.77 | 0.08 | 0.55 | 0.16 | 0.52 |
| Total bilirubin (µmol/L) | Too many values < LLOQ | | | | | | | | | | |
| Creatinine (µmol/L) | 44.7 | 9.3 | 43.0^b^ | 7.3 | 56.5 | 3.7 | 59.2 | 10.6 | <0.001 | 0.89 | 0.54 |
| Urea (mmol/L) | 2.88 | 1.02 | 2.20 | 0.80 | 2.20 | 0.60 | 1.83 | 0.41 | 0.13 | 0.09 | 0.67 |
| HDL (mmol/L) | 1.66 | 0.81 | 1.80 | 0.63 | 1.23 | 0.12 | 1.04 | 0.22 | <0.05 | 0.90 | 0.27 |
| Non-HDL (mmol/L) | 1.59^aa^ | 0.54 | 1.80^bb^ | 0.50 | 0.75 | 0.21 | 0.83 | 0.24 | <0.001 | 0.39 | 0.70 |
| HDL/non-HDL ratio | 1.01^a^ | 0.25 | 1.02 | 0.31 | 1.78 | 0.56 | 1.30 | 0.19 | <0.01 | 0.22 | 0.32 |
| TG (mmol/L) | 0.7 | 0.2 | 0.6 | 0.2 | 0.8 | 0.4 | 0.6 | 0.2 | 0.91 | 0.16 | 0.74 |
| Total cholesterol (mmol/L) | 4.0^a^ | 1.5 | 4.1^b^ | 1.1 | 2.3 | 0.3 | 2.3 | 0.5 | <0.001 | 0.81 | 0.74 |
| 3-HB (umol/L) | 16.8 | 13.0 | 17.0 | 7.7 | 10.3 | 4.5 | 12.0 | 4.6 | 0.14 | 0.43 | 0.91 |
| Fructosamine (µmol/L) | 215.5 | 13.8 | 214.2 | 9.2 | 209.2 | 31.1 | 201.8 | 17.9 | 0.28 | 0.62 | 0.72 |
| FFA (mmol/L) | 0.23 | 0.10 | 0.17 | 0.05 | 0.27 | 0.15 | 0.31 | 0.16 | 0.18 | 0.79 | 0.41 |
| GLY (mmol/L) | 0.04^a^ | 0.01 | 0.04 | 0.01 | 0.02 | 0.01 | 0.02 | 0.01 | <0.01 | 0.57 | 0.57 |
| ALB (g/L) | 39.6 | 3.9 | 41.3 | 4.6 | 40.9 | 6.3 | 38.6 | 2.8 | 0.72 | 0.91 | 0.30 |
| Haptoglobin (g/L) | 4.2^a^ | 1.9 | 3.3 | 1.4 | 2.2 | 0.4 | 2.2 | 0.4 | <0.01 | 0.45 | 0.40 |
| CRP (µg/mL) | 82.8 | 47.7 | 92.9 | 73.4 | 52.9 | 20.2 | 56.0 | 30.3 | 0.15 | 0.89 | 0.96 |
| BCAA (µM) | 775.2 | 164.3 | 836.4 | 158.8 | 626.8 | 149.1 | 575.3 | 115.5 | <0.05 | 0.85 | 0.59 |
| **Group** | **Females HFD** | | **Males HFD** | | **Females chow** | | **Males chow** | | **Two-way ANOVA p-values** | | |
| **Parameter** | **Mean** | **SD** | **Mean** | **SD** | **Mean** | **SD** | **Mean** | **SD** | **Diet** | **Sex** | **Sex*diet** |
| Total AA (µM) | 4258.9 | 706.9 | 5105.3 | 859.8 | 4486.8 | 600.2 | 4254.3 | 339.3 | 0.40 | 0.40 | 0.15 |
| ImP (nM) | 22.1 | 14.7 | 14.1^bb^ | 8.4 | 26.3 | 10.3 | 37.6 | 6.9 | <0.01 | 0.67 | <0.05 |
| Urocanate (nM) | 85.1 | 32.0 | 82.1 | 4.3 | 106.5 | 63.1 | 95.6 | 39.7 | 0.66 | 0.72 | 0.83 |
| Formic acid (µM) | 310.8 | 45.2 | 386.0^b^ | 48.1 | 301.2 | 35.3 | 301.9 | 57.8 | <0.05 | 0.07 | 0.07 |
| Acetic acid (µM) | 71.3^aa^ | 30.2 | 82.6^bb^ | 37.6 | 178.8 | 60.7 | 192.9 | 23.7 | <0.001 | 0.47 | 0.94 |
| Propionic acid (µM) | 0.77^a^ | 0.31 | 0.86 | 0.42 | 1.79 | 0.96 | 1.43 | 0.46 | <0.01 | 0.90 | 0.57 |
| Butyric acid (µM) | 1.16^aaa^ | 0.57 | 1.52^bb^ | 0.25 | 3.89 | 2.17 | 4.38 | 1.91 | <0.001 | 0.15 | 0.61 |
| Isobutyric acid (µM) | 0.30 | 0.12 | 0.28 | 0.06 | 0.43 | 0.14 | 0.50 | 0.16 | <0.01 | 0.61 | 0.61 |
| Succinic acid (µM) | 4.94 | 0.64 | 4.84 | 0.98 | 6.24 | 2.70 | 7.71 | 4.17 | 0.12 | 0.74 | 0.46 |
| Valeric acid (µM) | 0.13 | 0.05 | 0.11 | 0.07 | 0.12 | 0.06 | 0.13 | 0.05 | 0.83 | 0.63 | 0.55 |
| Isovaleric acid (µM) | 0.09^aa^ | 0.02 | 0.08^bb^ | 0.02 | 0.23 | 0.09 | 0.21 | 0.07 | <0.001 | 0.48 | 0.77 |
| Caprionic acid (µM) | Too many values < LLOQ | | | | | | | | | | |
| Calcium (mmol/L) | 2.65 | 0.13 | 2.74 | 0.06 | 2.72 | 0.07 | 2.67 | 0.15 | 0.97 | 0.67 | 0.15 |
| Magnesium (mmol/L) | 0.84 | 0.13 | 0.88 | 0.07 | 0.95 | 0.13 | 0.90 | 0.16 | 0.29 | 0.94 | 0.41 |
| Phosphorus (mmol/L) | 2.47 | 0.36 | 2.54 | 0.18 | 2.50 | 0.31 | 2.31 | 0.25 | 0.42 | 0.62 | 0.28 |
| Sodium (mmol/L) | 136.5 | 4.4 | 137.0 | 2.0 | 138.8 | 2.0 | 143.2 | 5.6 | p<0.05 | 0.15 | 0.25 |
| Potassium (mmol/L) | 4.38 | 0.32 | 4.44 | 0.12 | 4.62 | 0.77 | 4.69 | 0.44 | 0.25 | 0.77 | 0.98 |
| Chloride (mmol/L) | 96.3 | 5.2 | 96.6 | 2.9 | 95.6 | 1.1 | 97.1 | 2.6 | 0.94 | 0.52 | 0.68 |
| Total Protein (g/L) | 64.82 | 5.02 | 63.24 | 1.05 | 62.17 | 2.64 | 61.60 | 1.85 | 0.12 | 0.45 | 0.74 |
