# Supplementary material for: High fat diet is associated with gut microbiota dysbiosis and decreased gut microbial derived metabolites related to metabolic health in young Göttingen Minipigs
Source: PLoS One. 2024 Mar 1;19(3):e0298602. doi: 10.1371/journal.pone.0298602 (PMC10906878; doi:10.1371/journal.pone.0298602)

Supplementary Table S4

1. Bacterial taxa at family level significantly different in abundance between test diets having sex as cofactor. DESeq2 wrapped in DAtest package was used. Table includes bacterial families with log Fold Change above 2 or below -2 and an adjusted p-value < 0.05 was considered significantly different between the groups. HFD: high fat diet.

| Feature | Ordering | pval.adj | Family |
| --- | --- | --- | --- |
| zOTU_724 | Chow>HFD | 0.02784968 | f__Fibrobacteraceae |
| zOTU_914 | Chow>HFD | 0.00629365 | f__Flavobacteriaceae |
| zOTU_438 | Chow>HFD | 0.01731225 | f__Porphyromonadaceae |
| zOTU_1384 | Chow>HFD | 0.00159976 | f__Sphingobacteriaceae |
| zOTU_41 | Chow>HFD | 0.00381457 | f__Streptococcaceae |
| zOTU_1420 | HFD>Chow | 0.01992268 | f__Bacteroidaceae |
| zOTU_2 | HFD>Chow | 0.00077212 | f__Clostridiaceae_1 |
| zOTU_47 | HFD>Chow | 0.02857361 | f__Enterobacteriaceae |
| zOTU_478 | HFD>Chow | 0.02784968 | f__Eubacteriaceae |

1. Bacterial taxa at family level significantly different between treatment diets. Diet is included as class and sex as subclass using Huttenhower LEfSe online tool with default settings. Kruskal-Wallis test for classes reached significance when *P*<0.05 and 2.5 for threshold on the logarithmic LDA score for discriminative features. LDA scores were exported from https://huttenhower.sph.harvard.edu/galaxy/ and plotted R software v4.1.1. Bacteria families associated with Chow are colored green. while those associated with HFD are colored red.


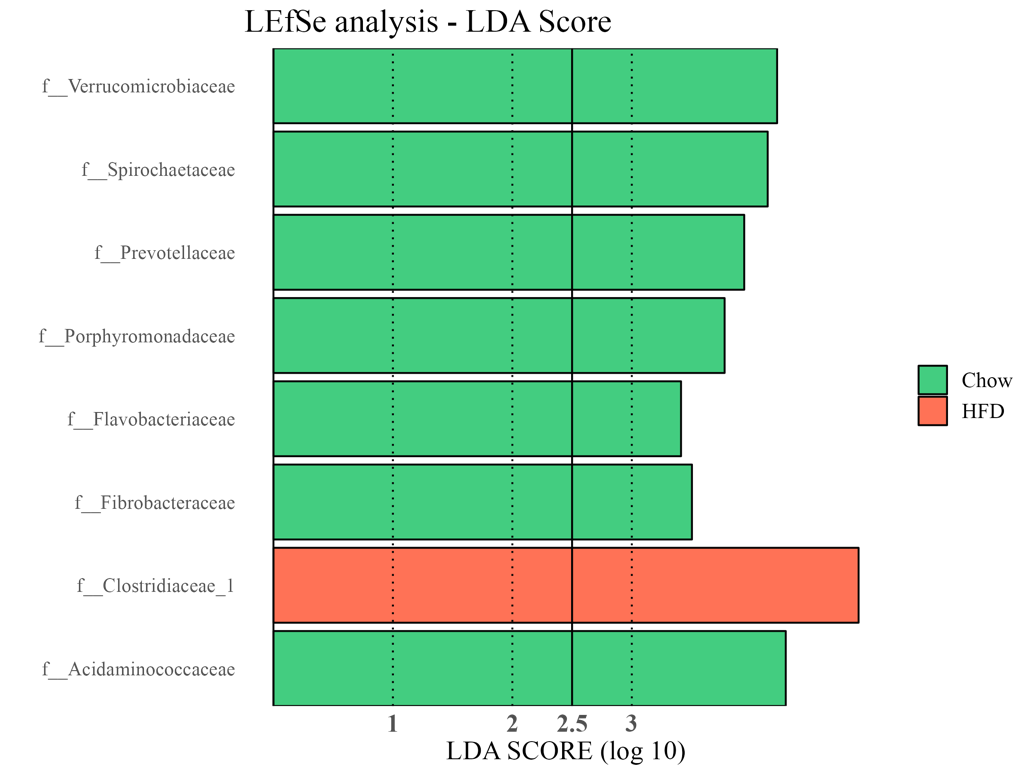

Supplement: S4 Table — A. Bacterial taxa at family level significantly different between test diets having sex as cofactor. DESeq2 wrapped in DAtest package was used. Table includes bacterial families with log Fold Change above 2 or below -2 and an adjusted p-value < 0.05 was considered significantly different between the groups. OUT: operational taxonomic units, HFD: high fat diet. B. Bacterial taxa at family level significantly different between treatment diets. Diet is included as class and sex as subclass using Huttenhower LEfSe online tool with default settings. Kruskal-Wallis test for classes reached significance when p < 0.05 and 2.5 for threshold on the logarithmic LDA score for discriminative features. Picture exported from https://huttenhower.sph.harvard.edu/galaxy/. (DOCX) [file pone.0298602.s009.docx]
