# Supplementary material for: High fat diet is associated with gut microbiota dysbiosis and decreased gut microbial derived metabolites related to metabolic health in young Göttingen Minipigs
Source: PLoS One. 2024 Mar 1;19(3):e0298602. doi: 10.1371/journal.pone.0298602 (PMC10906878; doi:10.1371/journal.pone.0298602)

**Supplementary table S5**

1. Bacterial taxa significantly different between treatment diets having sex as covariate and mapped to taxonomical level genus or species. DESeq2 wrapped in DAtest r package was used. Table includes log Fold Change above 2 or below -2. OTUs mapped at species level and an adjusted p-value < 0.05 was considered significantly different. NA: Not available.

| **Feature** | **log2**  **FoldChange** | | **Accociated with** | **pval.adj** | **Family** | **Genus** | **Species** |
| --- | --- | --- | --- | --- | --- | --- | --- |
| zOTU_554 | | -2.2113213 | Chow>HFD | 0.01672716 | f__Lachnospiraceae | g__Clostridium_XlVa | s__Clostridium_aerotolerans |
| zOTU_449 | | -2.0325931 | Chow>HFD | 0.03059576 | f__Lachnospiraceae | g__Clostridium_XlVa | s__Clostridium_aerotolerans |
| zOTU_1028 | | -2.8010005 | Chow>HFD | 0.03333839 | f__Ruminococcaceae | g__Clostridium_IV | s__Clostridium_leptum |
| zOTU_483 | | -5.4806314 | Chow>HFD | 0.00011693 | f__Lachnospiraceae | g__Lachnospiracea_incertae_sedis | s__Eubacterium_eligens |
| zOTU_465 | | -5.2164161 | Chow>HFD | 0.00013247 | f__Lachnospiraceae | g__Lachnospiracea_incertae_sedis | s__Eubacterium_eligens |
| zOTU_373 | | -5.1240824 | Chow>HFD | 0.00013693 | f__Lachnospiraceae | g__Lachnospiracea_incertae_sedis | s__Eubacterium_eligens |
| zOTU_534 | | -5.0771134 | Chow>HFD | 0.00076588 | f__Lachnospiraceae | g__Lachnospiracea_incertae_sedis | s__Eubacterium_eligens |
| zOTU_414 | | -3.1832254 | Chow>HFD | 0.00179705 | f__Lachnospiraceae | g__Lachnospiracea_incertae_sedis | s__Eubacterium_eligens |
| zOTU_427 | | -3.1925139 | Chow>HFD | 0.0032621 | f__Lachnospiraceae | g__Lachnospiracea_incertae_sedis | s__Eubacterium_eligens |
| zOTU_338 | | -3.103912 | Chow>HFD | 0.00435609 | f__Lachnospiraceae | g__Lachnospiracea_incertae_sedis | s__Eubacterium_eligens |
| zOTU_442 | | -3.1552673 | Chow>HFD | 0.0059597 | f__Lachnospiraceae | g__Lachnospiracea_incertae_sedis | s__Eubacterium_eligens |
| zOTU_966 | | -2.8616805 | Chow>HFD | 0.00371118 | f__Ruminococcaceae | g__Clostridium_IV | s__Eubacterium_siraeum |
| zOTU_367 | | -3.3525403 | Chow>HFD | 0.00461204 | f__Ruminococcaceae | g__Clostridium_IV | s__Eubacterium_siraeum |
| zOTU_295 | | -3.08748 | Chow>HFD | 0.004989 | f__Ruminococcaceae | g__Clostridium_IV | s__Eubacterium_siraeum |
| zOTU_404 | | -3.4753376 | Chow>HFD | 0.00755878 | f__Ruminococcaceae | g__Clostridium_IV | s__Eubacterium_siraeum |
| zOTU_377 | | -2.9938527 | Chow>HFD | 0.00817639 | f__Ruminococcaceae | g__Clostridium_IV | s__Eubacterium_siraeum |
| zOTU_937 | | -3.3722575 | Chow>HFD | 0.00024478 | f__Ruminococcaceae | g__Faecalibacterium | s__Faecalibacterium_prausnitzii |
| zOTU_1044 | | -2.4963951 | Chow>HFD | 0.00995043 | f__Ruminococcaceae | g__Faecalibacterium | s__Faecalibacterium_prausnitzii |
| zOTU_865 | | -3.2914936 | Chow>HFD | 0.00076588 | f__Fibrobacteraceae | g__Fibrobacter | s__Fibrobacter_intestinalis |
| zOTU_939 | | -2.4026145 | Chow>HFD | 0.01316027 | f__Fibrobacteraceae | g__Fibrobacter | s__Fibrobacter_intestinalis |
| zOTU_724 | | -2.3199967 | Chow>HFD | 0.0416182 | f__Fibrobacteraceae | g__Fibrobacter | s__Fibrobacter_intestinalis |
| zOTU_900 | | -2.000392 | Chow>HFD | 0.0454542 | f__Fibrobacteraceae | g__Fibrobacter | s__Fibrobacter_intestinalis |
| zOTU_908 | | -3.1993255 | Chow>HFD | 0.02878126 | f__Ruminococcaceae | g__Flavonifractor | s__Flavonifractor_plautii |
| zOTU_461 | | -3.6265698 | Chow>HFD | 0.00461204 | f__Ruminococcaceae | g__Gemmiger | s__Gemmiger_formicilis |
| zOTU_388 | | -2.7348503 | Chow>HFD | 0.01949328 | f__Ruminococcaceae | g__Gemmiger | s__Gemmiger_formicilis |
| zOTU_462 | | -3.4743572 | Chow>HFD | 0.0203141 | f__Ruminococcaceae | g__Gemmiger | s__Gemmiger_formicilis |
| zOTU_472 | | -2.4466051 | Chow>HFD | 0.01563962 | f__Erysipelotrichaceae | g__Holdemanella | s__Holdemanella_biformis |
| zOTU_457 | | -2.4442442 | Chow>HFD | 0.02268124 | f__Erysipelotrichaceae | g__Holdemanella | s__Holdemanella_biformis |
| zOTU_463 | | -2.3285503 | Chow>HFD | 0.02826723 | f__Erysipelotrichaceae | g__Holdemanella | s__Holdemanella_biformis |
| zOTU_371 | | -2.307462 | Chow>HFD | 0.03220448 | f__Erysipelotrichaceae | g__Holdemanella | s__Holdemanella_biformis |
| zOTU_79 | | -2.004005 | Chow>HFD | 0.02106529 | f__Ruminococcaceae | g__Intestinimonas | s__Intestinimonas_butyriciproducens |
| zOTU_350 | | -3.1054907 | Chow>HFD | 0.01505397 | f__Acidaminococcaceae | g__Phascolarctobacterium | s__Phascolarctobacterium_succinatutens |
| zOTU_324 | | -2.7344545 | Chow>HFD | 0.02186827 | f__Acidaminococcaceae | g__Phascolarctobacterium | s__Phascolarctobacterium_succinatutens |
| zOTU_340 | | -2.7087519 | Chow>HFD | 0.02745869 | f__Acidaminococcaceae | g__Phascolarctobacterium | s__Phascolarctobacterium_succinatutens |
| zOTU_248 | | -2.6767591 | Chow>HFD | 0.03264589 | f__Acidaminococcaceae | g__Phascolarctobacterium | s__Phascolarctobacterium_succinatutens |
| zOTU_86 | | -2.5249529 | Chow>HFD | 0.04274233 | f__Acidaminococcaceae | g__Phascolarctobacterium | s__Phascolarctobacterium_succinatutens |
| zOTU_101 | | -2.4975966 | Chow>HFD | 0.04284307 | f__Acidaminococcaceae | g__Phascolarctobacterium | s__Phascolarctobacterium_succinatutens |
| zOTU_923 | | -3.8326388 | Chow>HFD | 0.00039373 | f__Prevotellaceae | g__Prevotella | s__Prevotella_dentasini |
| zOTU_885 | | -3.9611544 | Chow>HFD | 0.00168085 | f__Prevotellaceae | g__Prevotella | s__Prevotella_dentasini |
| zOTU_728 | | -3.4972836 | Chow>HFD | 0.00224792 | f__Prevotellaceae | g__Prevotella | s__Prevotella_dentasini |
| zOTU_890 | | -3.6529132 | Chow>HFD | 0.002302 | f__Prevotellaceae | g__Prevotella | s__Prevotella_dentasini |
| zOTU_946 | | -5.8046005 | Chow>HFD | 0.00016837 | f__Ruminococcaceae | g__Ruminococcus | s__Ruminococcus_bromii |
| zOTU_601 | | -3.7792004 | Chow>HFD | 0.00076588 | f__Spirochaetaceae | g__Treponema | s__Treponema_berlinense |
| zOTU_571 | | -3.3532248 | Chow>HFD | 0.00169416 | f__Spirochaetaceae | g__Treponema | s__Treponema_berlinense |
| zOTU_570 | | -3.1779143 | Chow>HFD | 0.00198247 | f__Spirochaetaceae | g__Treponema | s__Treponema_berlinense |
| zOTU_453 | | -3.3643039 | Chow>HFD | 0.00374822 | f__Spirochaetaceae | g__Treponema | s__Treponema_berlinense |
| zOTU_349 | | -3.1549659 | Chow>HFD | 0.01949328 | f__Spirochaetaceae | g__Treponema | s__Treponema_bryantii |
| zOTU_325 | | -2.863552 | Chow>HFD | 0.03322947 | f__Spirochaetaceae | g__Treponema | s__Treponema_bryantii |
| zOTU_339 | | -2.6380884 | Chow>HFD | 0.04932469 | f__Spirochaetaceae | g__Treponema | s__Treponema_bryantii |
| zOTU_1037 | | -5.1842357 | Chow>HFD | 3.0491E-05 | f__Prevotellaceae | g__Prevotella | NA |
| zOTU_931 | | -5.147428 | Chow>HFD | 3.0491E-05 | f__Prevotellaceae | g__Prevotella | NA |
| zOTU_418 | | -3.9927862 | Chow>HFD | 0.00014144 | f__Prevotellaceae | g__Prevotella | NA |
| zOTU_379 | | -4.1365178 | Chow>HFD | 0.00016837 | f__Prevotellaceae | g__Prevotella | NA |
| zOTU_920 | | -4.5586782 | Chow>HFD | 0.00020192 | f__Ruminococcaceae | g__Ruminococcus | NA |
| zOTU_897 | | -4.9261032 | Chow>HFD | 0.0002064 | f__Ruminococcaceae | g__Ruminococcus | NA |
| zOTU_781 | | -4.7353312 | Chow>HFD | 0.00024562 | f__Ruminococcaceae | g__Ruminococcus | NA |
| zOTU_918 | | -5.2726681 | Chow>HFD | 0.00033845 | f__Lachnospiraceae | g__Clostridium_XlVa | NA |
| zOTU_1025 | | -4.1461619 | Chow>HFD | 0.00039373 | f__Prevotellaceae | g__Prevotella | NA |
| zOTU_1038 | | -3.9961594 | Chow>HFD | 0.00039373 | f__Lachnospiraceae | g__Blautia | NA |
| zOTU_872 | | -4.3862549 | Chow>HFD | 0.00044325 | f__Prevotellaceae | g__Prevotella | NA |
| zOTU_1011 | | -3.0852305 | Chow>HFD | 0.00076613 | f__Prevotellaceae | g__Prevotella | NA |
| zOTU_745 | | -5.2232653 | Chow>HFD | 0.00076613 | f__Lachnospiraceae | g__Clostridium_XlVa | NA |
| zOTU_821 | | -4.0966 | Chow>HFD | 0.00076613 | f__Prevotellaceae | g__Prevotella | NA |
| zOTU_1012 | | -2.6994816 | Chow>HFD | 0.00085204 | f__Prevotellaceae | g__Prevotella | NA |
| zOTU_402 | | -3.3830857 | Chow>HFD | 0.00085204 | f__Prevotellaceae | g__Prevotella | NA |
| zOTU_320 | | -3.5863627 | Chow>HFD | 0.00102578 | f__Prevotellaceae | g__Prevotella | NA |
| zOTU_924 | | -2.8667053 | Chow>HFD | 0.00102578 | f__Prevotellaceae | g__Prevotella | NA |
| zOTU_857 | | -5.0949105 | Chow>HFD | 0.00110147 | f__Lachnospiraceae | g__Clostridium_XlVa | NA |
| zOTU_44 | | -5.8120688 | Chow>HFD | 0.00154976 | f__Streptococcaceae | g__Streptococcus | NA |
| zOTU_51 | | -5.8221627 | Chow>HFD | 0.00154976 | f__Streptococcaceae | g__Streptococcus | NA |
| zOTU_928 | | -4.2433144 | Chow>HFD | 0.0015964 | f__Prevotellaceae | g__Prevotella | NA |
| zOTU_41 | | -6.1525306 | Chow>HFD | 0.00162793 | f__Streptococcaceae | g__Streptococcus | NA |
| zOTU_954 | | -4.1580169 | Chow>HFD | 0.00165075 | f__Prevotellaceae | g__Prevotella | NA |
| zOTU_758 | | -4.0383596 | Chow>HFD | 0.00168085 | f__Prevotellaceae | g__Prevotella | NA |
| zOTU_651 | | -3.6101687 | Chow>HFD | 0.00186695 | f__Prevotellaceae | g__Prevotella | NA |
| zOTU_853 | | -2.6223312 | Chow>HFD | 0.00236442 | f__Prevotellaceae | g__Prevotella | NA |
| zOTU_48 | | -5.4905078 | Chow>HFD | 0.00277378 | f__Streptococcaceae | g__Streptococcus | NA |
| zOTU_1000 | | -4.064742 | Chow>HFD | 0.00286839 | f__Ruminococcaceae | g__Ruminococcus | NA |
| zOTU_592 | | -2.9782229 | Chow>HFD | 0.00333031 | f__Prevotellaceae | g__Prevotella | NA |
| zOTU_1026 | | -5.2585065 | Chow>HFD | 0.00357222 | f__Lachnospiraceae | g__Clostridium_XlVa | NA |
| zOTU_669 | | -3.134445 | Chow>HFD | 0.00358688 | f__Prevotellaceae | g__Prevotella | NA |
| zOTU_983 | | -3.7017186 | Chow>HFD | 0.00551413 | f__Prevotellaceae | g__Prevotella | NA |
| zOTU_495 | | -2.9846755 | Chow>HFD | 0.00572414 | f__Prevotellaceae | g__Prevotella | NA |
| zOTU_679 | | -3.4024151 | Chow>HFD | 0.00661257 | f__Prevotellaceae | g__Prevotella | NA |
| zOTU_889 | | -2.9006614 | Chow>HFD | 0.00956917 | f__Prevotellaceae | g__Prevotella | NA |
| zOTU_842 | | -4.2411607 | Chow>HFD | 0.00992443 | f__Lachnospiraceae | g__Clostridium_XlVa | NA |
| zOTU_895 | | -2.623179 | Chow>HFD | 0.01165732 | f__Prevotellaceae | g__Prevotella | NA |
| zOTU_739 | | -2.8306169 | Chow>HFD | 0.01193258 | f__Prevotellaceae | g__Prevotella | NA |
| zOTU_858 | | -3.3561438 | Chow>HFD | 0.012184 | f__Prevotellaceae | g__Prevotella | NA |
| zOTU_906 | | -4.0571059 | Chow>HFD | 0.01221334 | f__Lachnospiraceae | g__Clostridium_XlVa | NA |
| zOTU_1034 | | -4.198078 | Chow>HFD | 0.01237828 | f__Lachnospiraceae | g__Clostridium_XlVa | NA |
| zOTU_967 | | -2.6865678 | Chow>HFD | 0.01515471 | f__Prevotellaceae | g__Prevotella | NA |
| zOTU_401 | | -4.611782 | Chow>HFD | 0.01949328 | f__Streptococcaceae | g__Streptococcus | NA |
| zOTU_1024 | | -3.7927103 | Chow>HFD | 0.02185082 | f__Lachnospiraceae | g__Clostridium_XlVa | NA |
| zOTU_448 | | -4.0531008 | Chow>HFD | 0.02846406 | f__Streptococcaceae | g__Streptococcus | NA |
| zOTU_494 | | -4.3794145 | Chow>HFD | 0.03333839 | f__Streptococcaceae | g__Streptococcus | NA |
| zOTU_985 | | -2.1728623 | Chow>HFD | 0.03804607 | f__Lachnospiraceae | g__Clostridium_XlVa | NA |
| zOTU_1019 | | -2.4176938 | Chow>HFD | 0.04063832 | f__Prevotellaceae | g__Prevotella | NA |
| zOTU_852 | | 4.10385808 | HFD>Chow | 0.00032623 | f__Lachnospiraceae | g__Cellulosilyticum | s__Cellulosilyticum_ruminicola |
| zOTU_1031 | | 4.20089926 | HFD>Chow | 0.00044325 | f__Lachnospiraceae | g__Cellulosilyticum | s__Cellulosilyticum_ruminicola |
| zOTU_798 | | 23.4130528 | HFD>Chow | 3.6178E-17 | f__Eubacteriaceae | g__Eubacterium | s__Eubacterium_coprostanoligenes |
| zOTU_575 | | 9.41345362 | HFD>Chow | 0.0002064 | f__Eubacteriaceae | g__Eubacterium | s__Eubacterium_coprostanoligenes |
| zOTU_477 | | 8.1156403 | HFD>Chow | 0.00020752 | f__Eubacteriaceae | g__Eubacterium | s__Eubacterium_coprostanoligenes |
| zOTU_641 | | 7.46104424 | HFD>Chow | 0.00039373 | f__Eubacteriaceae | g__Eubacterium | s__Eubacterium_coprostanoligenes |
| zOTU_649 | | 7.25709791 | HFD>Chow | 0.00133092 | f__Eubacteriaceae | g__Eubacterium | s__Eubacterium_coprostanoligenes |
| zOTU_1046 | | 6.89152266 | HFD>Chow | 0.00181905 | f__Eubacteriaceae | g__Eubacterium | s__Eubacterium_coprostanoligenes |
| zOTU_1005 | | 6.32574776 | HFD>Chow | 0.00611536 | f__Eubacteriaceae | g__Eubacterium | s__Eubacterium_coprostanoligenes |
| zOTU_936 | | 6.00691758 | HFD>Chow | 0.00992443 | f__Eubacteriaceae | g__Eubacterium | s__Eubacterium_coprostanoligenes |
| zOTU_631 | | 2.98001339 | HFD>Chow | 0.00872709 | f__Lactobacillaceae | g__Lactobacillus | s__Lactobacillus_equicursoris |
| zOTU_717 | | 2.92962044 | HFD>Chow | 0.02341503 | f__Lactobacillaceae | g__Lactobacillus | s__Lactobacillus_equicursoris |
| zOTU_52 | | 2.31117303 | HFD>Chow | 0.00415344 | f__Lactobacillaceae | g__Lactobacillus | s__Lactobacillus_mucosae |
| zOTU_75 | | 2.28746125 | HFD>Chow | 0.004989 | f__Lactobacillaceae | g__Lactobacillus | s__Lactobacillus_mucosae |
| zOTU_78 | | 2.19639077 | HFD>Chow | 0.00543831 | f__Lactobacillaceae | g__Lactobacillus | s__Lactobacillus_mucosae |
| zOTU_67 | | 2.12988548 | HFD>Chow | 0.00995043 | f__Lactobacillaceae | g__Lactobacillus | s__Lactobacillus_mucosae |
| zOTU_281 | | 6.55615068 | HFD>Chow | 0.00198247 | f__Acidaminococcaceae | g__Phascolarctobacterium | s__Phascolarctobacterium_succinatutens |
| zOTU_506 | | 5.68445508 | HFD>Chow | 0.00520355 | f__Acidaminococcaceae | g__Phascolarctobacterium | s__Phascolarctobacterium_succinatutens |
| zOTU_202 | | 6.04807157 | HFD>Chow | 0.00852284 | f__Acidaminococcaceae | g__Phascolarctobacterium | s__Phascolarctobacterium_succinatutens |
| zOTU_215 | | 6.19888071 | HFD>Chow | 0.0094441 | f__Acidaminococcaceae | g__Phascolarctobacterium | s__Phascolarctobacterium_succinatutens |
| zOTU_272 | | 5.90887529 | HFD>Chow | 0.00992443 | f__Acidaminococcaceae | g__Phascolarctobacterium | s__Phascolarctobacterium_succinatutens |
| zOTU_549 | | 5.28062114 | HFD>Chow | 0.01190093 | f__Acidaminococcaceae | g__Phascolarctobacterium | s__Phascolarctobacterium_succinatutens |
| zOTU_294 | | 5.8347611 | HFD>Chow | 0.01217454 | f__Acidaminococcaceae | g__Phascolarctobacterium | s__Phascolarctobacterium_succinatutens |
| zOTU_252 | | 5.89672752 | HFD>Chow | 0.01454375 | f__Acidaminococcaceae | g__Phascolarctobacterium | s__Phascolarctobacterium_succinatutens |
| zOTU_329 | | 5.83996695 | HFD>Chow | 0.02180872 | f__Acidaminococcaceae | g__Phascolarctobacterium | s__Phascolarctobacterium_succinatutens |
| zOTU_381 | | 5.28707556 | HFD>Chow | 0.0232558 | f__Acidaminococcaceae | g__Phascolarctobacterium | s__Phascolarctobacterium_succinatutens |
| zOTU_300 | | 5.75717782 | HFD>Chow | 0.0259107 | f__Acidaminococcaceae | g__Phascolarctobacterium | s__Phascolarctobacterium_succinatutens |
| zOTU_469 | | 5.11164769 | HFD>Chow | 0.04267885 | f__Acidaminococcaceae | g__Phascolarctobacterium | s__Phascolarctobacterium_succinatutens |
| zOTU_82 | | 2.76353309 | HFD>Chow | 6.3101E-05 | f__Peptostreptococcaceae | g__Terrisporobacter | s__Terrisporobacter_glycolicus |
| zOTU_100 | | 2.77936807 | HFD>Chow | 0.00016837 | f__Peptostreptococcaceae | g__Terrisporobacter | s__Terrisporobacter_glycolicus |
| zOTU_92 | | 2.63478797 | HFD>Chow | 0.00016837 | f__Peptostreptococcaceae | g__Terrisporobacter | s__Terrisporobacter_glycolicus |
| zOTU_64 | | 2.60368732 | HFD>Chow | 0.0003738 | f__Peptostreptococcaceae | g__Terrisporobacter | s__Terrisporobacter_glycolicus |
| zOTU_650 | | 3.79998699 | HFD>Chow | 0.00136249 | f__Erysipelotrichaceae | g__Turicibacter | s__Turicibacter_sanguinis |
| zOTU_480 | | 3.6836278 | HFD>Chow | 0.00256106 | f__Erysipelotrichaceae | g__Turicibacter | s__Turicibacter_sanguinis |
| zOTU_574 | | 3.64529541 | HFD>Chow | 0.00534402 | f__Erysipelotrichaceae | g__Turicibacter | s__Turicibacter_sanguinis |
| zOTU_614 | | 3.44254164 | HFD>Chow | 0.00771806 | f__Erysipelotrichaceae | g__Turicibacter | s__Turicibacter_sanguinis |
| zOTU_797 | | 5.10916862 | HFD>Chow | 0.00011693 | f__Clostridiaceae_1 | g__Clostridium_sensu_stricto | NA |
| zOTU_545 | | 3.24646025 | HFD>Chow | 0.00076588 | f__Lachnospiraceae | g__Cellulosilyticum | NA |
| zOTU_561 | | 3.25612022 | HFD>Chow | 0.00076613 | f__Lachnospiraceae | g__Cellulosilyticum | NA |
| zOTU_489 | | 3.42053894 | HFD>Chow | 0.00085204 | f__Lachnospiraceae | g__Cellulosilyticum | NA |
| zOTU_434 | | 3.24332957 | HFD>Chow | 0.00093719 | f__Lachnospiraceae | g__Cellulosilyticum | NA |
| zOTU_859 | | 8.62155836 | HFD>Chow | 0.00102578 | f__Prevotellaceae | g__Prevotella | NA |
| zOTU_719 | | 5.96973529 | HFD>Chow | 0.002302 | f__Prevotellaceae | g__Prevotella | NA |
| zOTU_1036 | | 7.00492701 | HFD>Chow | 0.00320226 | f__Prevotellaceae | g__Prevotella | NA |
| zOTU_981 | | 6.93444399 | HFD>Chow | 0.00353971 | f__Prevotellaceae | g__Prevotella | NA |
| zOTU_822 | | 7.58260587 | HFD>Chow | 0.00398848 | f__Prevotellaceae | g__Prevotella | NA |
| zOTU_731 | | 5.94420194 | HFD>Chow | 0.00469924 | f__Prevotellaceae | g__Prevotella | NA |
| zOTU_979 | | 7.29973228 | HFD>Chow | 0.004989 | f__Prevotellaceae | g__Prevotella | NA |
| zOTU_221 | | 2.96755734 | HFD>Chow | 0.00743943 | f__Lachnospiraceae | g__Blautia | NA |
| zOTU_257 | | 2.90267872 | HFD>Chow | 0.00817639 | f__Lachnospiraceae | g__Blautia | NA |
| zOTU_1045 | | 5.75546676 | HFD>Chow | 0.0082969 | f__Prevotellaceae | g__Prevotella | NA |
| zOTU_743 | | 5.65654968 | HFD>Chow | 0.00970133 | f__Prevotellaceae | g__Prevotella | NA |
| zOTU_809 | | 7.13311896 | HFD>Chow | 0.00992443 | f__Prevotellaceae | g__Prevotella | NA |
| zOTU_5 | | 2.13845757 | HFD>Chow | 0.01217454 | f__Clostridiaceae_1 | g__Clostridium_sensu_stricto | NA |
| zOTU_980 | | 6.4750526 | HFD>Chow | 0.01217454 | f__Prevotellaceae | g__Prevotella | NA |
| zOTU_2 | | 2.06828691 | HFD>Chow | 0.01366157 | f__Clostridiaceae_1 | g__Clostridium_sensu_stricto | NA |
| zOTU_7 | | 2.05829466 | HFD>Chow | 0.01563962 | f__Clostridiaceae_1 | g__Clostridium_sensu_stricto | NA |
| zOTU_176 | | 2.67116769 | HFD>Chow | 0.01604663 | f__Lachnospiraceae | g__Blautia | NA |
| zOTU_8 | | 2.04977846 | HFD>Chow | 0.01652891 | f__Clostridiaceae_1 | g__Clostridium_sensu_stricto | NA |
| zOTU_241 | | 2.78164804 | HFD>Chow | 0.01831236 | f__Lachnospiraceae | g__Blautia | NA |
| zOTU_915 | | 5.2491996 | HFD>Chow | 0.01949328 | f__Prevotellaceae | g__Prevotella | NA |
| zOTU_1018 | | 5.62639777 | HFD>Chow | 0.02106529 | f__Prevotellaceae | g__Prevotella | NA |
| zOTU_369 | | 2.74811598 | HFD>Chow | 0.02268124 | f__Lactobacillaceae | g__Lactobacillus | NA |
| zOTU_1030 | | 5.4794135 | HFD>Chow | 0.02606797 | f__Prevotellaceae | g__Prevotella | NA |
| zOTU_815 | | 5.02990769 | HFD>Chow | 0.02798199 | f__Lachnospiraceae | g__Clostridium_XlVa | NA |
| zOTU_607 | | 5.22318215 | HFD>Chow | 0.02944832 | f__Prevotellaceae | g__Prevotella | NA |
| zOTU_312 | | 2.55875 | HFD>Chow | 0.03222968 | f__Lactobacillaceae | g__Lactobacillus | NA |
| zOTU_447 | | 3.17153374 | HFD>Chow | 0.03546783 | f__Coriobacteriaceae | g__Olsenella | NA |
| zOTU_963 | | 4.15249519 | HFD>Chow | 0.0454542 | f__Lachnospiraceae | g__Clostridium_XlVa | NA |
| zOTU_929 | | 4.41596404 | HFD>Chow | 0.0485171 | f__Lachnospiraceae | g__Clostridium_XlVa | NA |


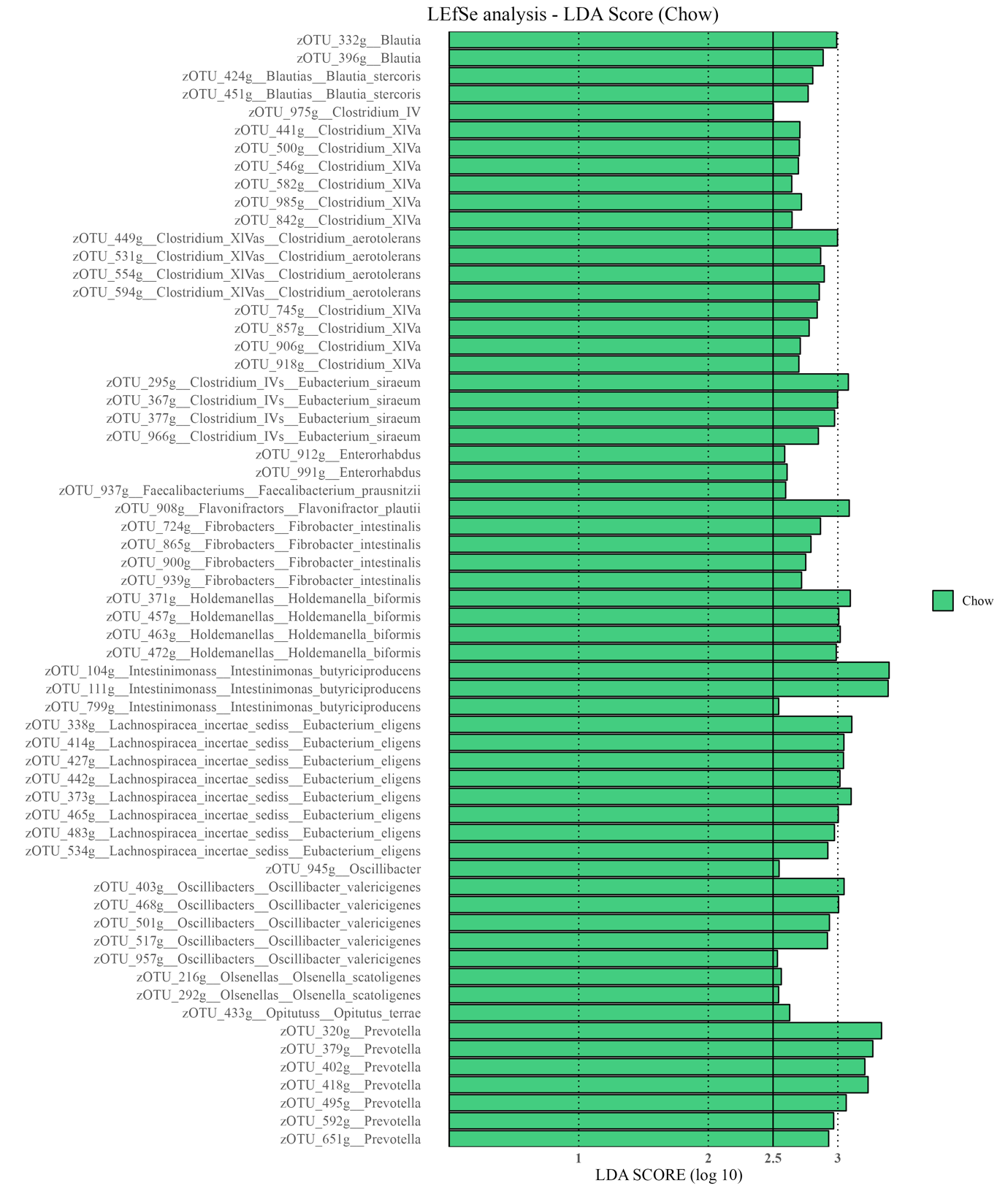
B) Significantly different OTU between treatment diets. Diet is included as class and sex as subclass using Huttenhower LEfSe online tool with default settings. Kruskal-Wallis test for classes and 2.5 for threshold on the logarithmic LDA score for discrimitive features. LDA scores exported from https://huttenhower.sph.harvard.edu/galaxy/ and plotted using R software v4.1.1. OUTs associated with Chow are colored green. while those associated with HFD are colored red.


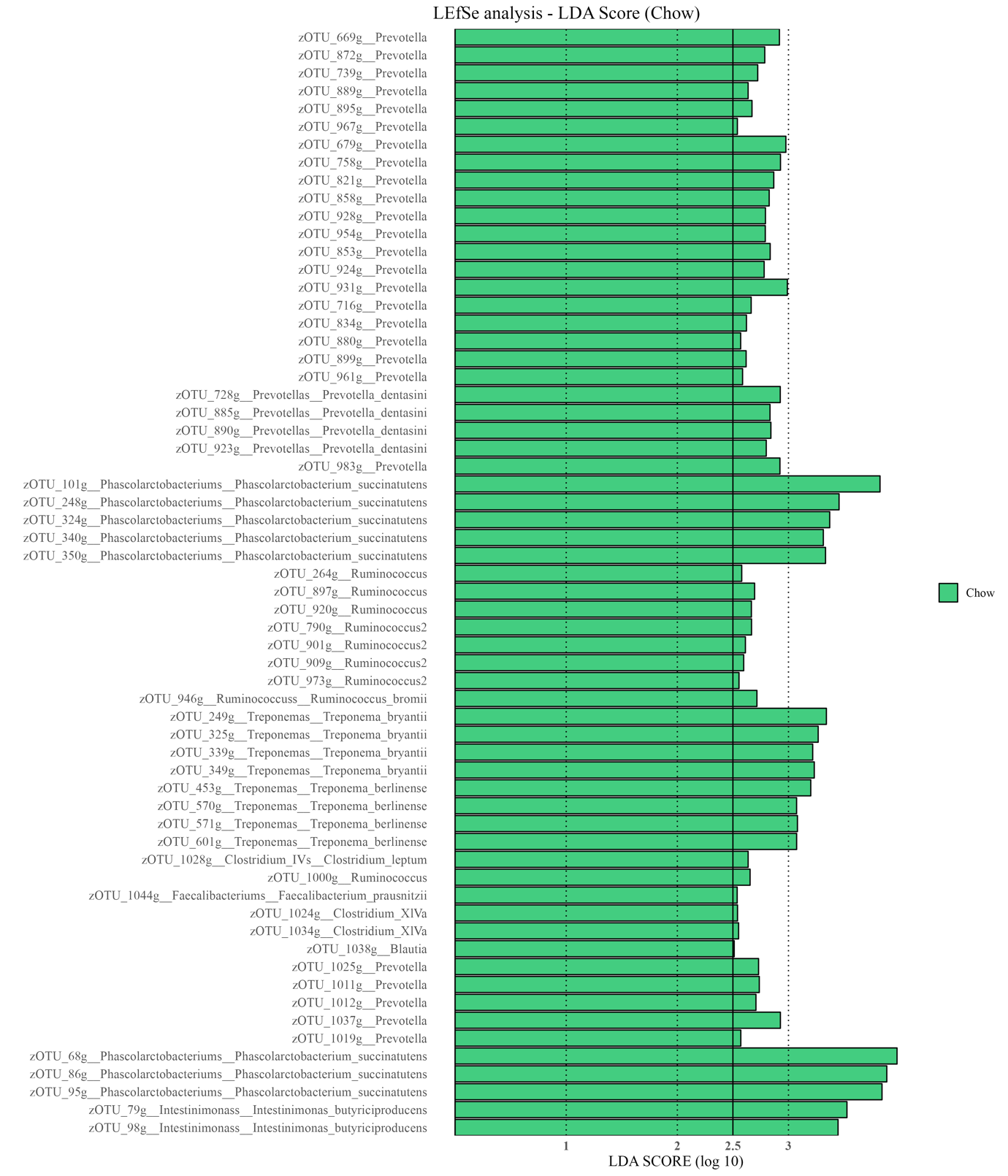


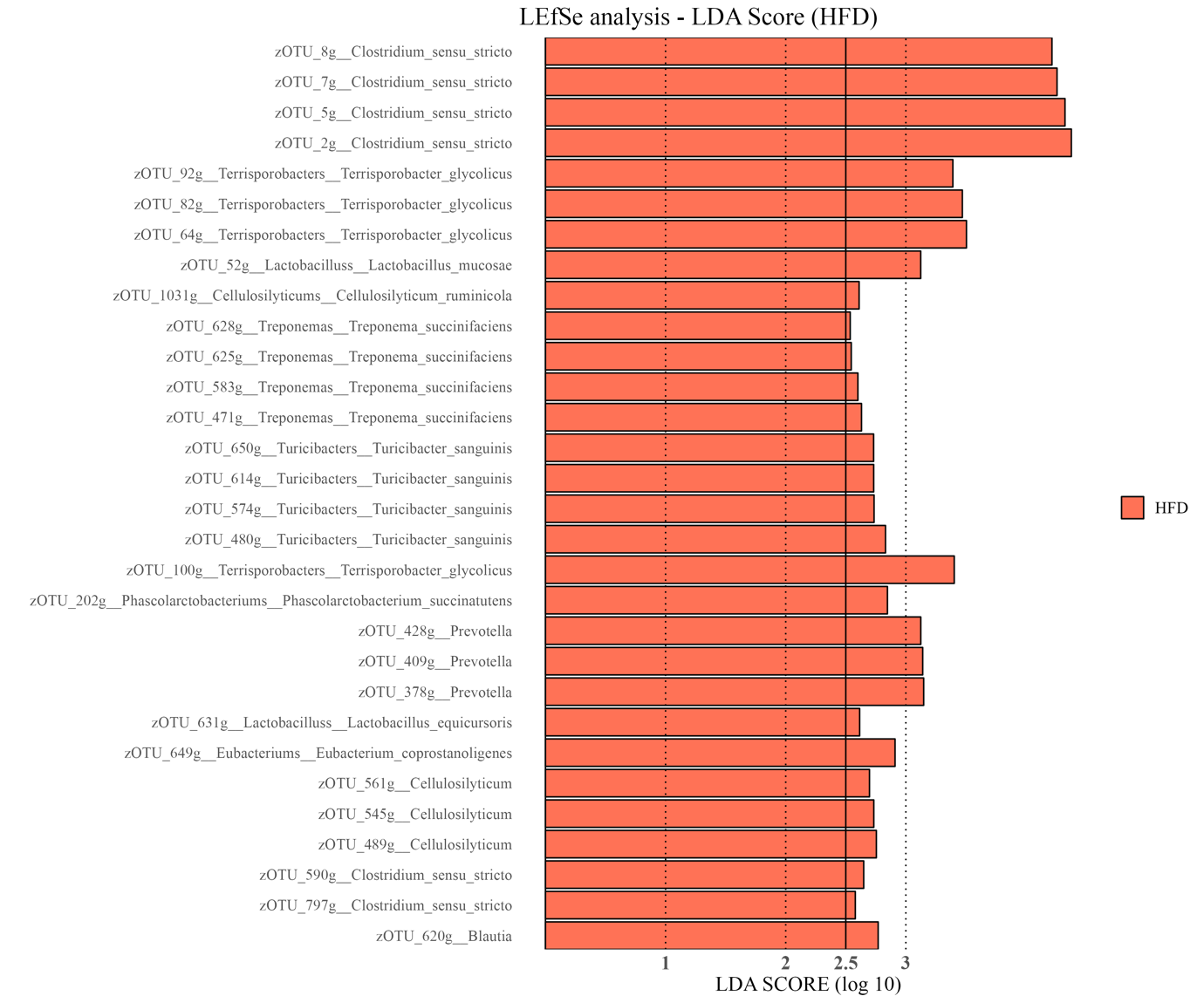

Supplement: S5 Table — A. Bacterial taxa significantly different between treatment diets having sex as covariate and mapped to taxonomical level genus or species. DESeq2 wrapped in DAtest r package was used. Table includes log Fold Change above 2 or below -2. OTUs mapped at species level and an adjusted p-value < 0.05 was considered significantly different. HFD: high fat diet. B. Significantly different OTU between treatment diets. Diet is included as class and sex as subclass using Huttenhower LEfSe online tool with default settings. Kruskal-Wallis test for classes and 2.5 for threshold on the logarithmic LDA score for discriminative features. Picture exported from https://huttenhower.sph.harvard.edu/galaxy/ and has been split into 5 images separate images (indicated in each split figure in the top right corner—x/5). (DOCX) [file pone.0298602.s010.docx]
