# Supplementary material for: High fat diet is associated with gut microbiota dysbiosis and decreased gut microbial derived metabolites related to metabolic health in young Göttingen Minipigs
Source: PLoS One. 2024 Mar 1;19(3):e0298602. doi: 10.1371/journal.pone.0298602 (PMC10906878; doi:10.1371/journal.pone.0298602)
Supplement: S9 Table — Body composition data and absolute and relative organ weights in castrated male (M) and ovariectomised female (F) Göttingen Minipigs fed chow or high fat diet (HFD). Two-way ANOVA with gender and diet as explanatory variables followed by Tukeys multiple comparison test. Only the following comparisons were considered relevant: M-HFD vs. M-chow, M-HFD vs. F-HFD, M-chow vs. F-chow and F-HFD vs. F-chow. * p<0.05, ** p<0.01, *** p<0.001. (DOCX) [file pone.0298602.s014.docx]

**S9 Table Body composition and organ weights**

Body composition data and absolute and relative organ weights in castrated male (M) and ovariectomised female (F) Göttingen Minipigs fed chow or high fat diet (HFD). Two-way ANOVA with gender and diet as explanatory variables followed by Tukeys multiple comparison test. Mean±SD, n=5-6. Only the following comparisons were considered relevant: M-HFD vs. M-chow, M-HFD vs. F-HFD, M-chow vs. F-chow and F-HFD vs. F-chow. Superscript numbers indicate significant difference between groups. ^aaa^ p<0.001 for F-HFD vs. F-chow, ^bbb^ p<0.001 for M-HFD vs. M-chow.

| **Group** | **Females HFD** | | **Males HFD** | | **Females chow** | | **Males chow** | | **Two-way ANOVA p-values** | | |
| --- | --- | --- | --- | --- | --- | --- | --- | --- | --- | --- | --- |
| **Parameter** | **Mean** | **SD** | **Mean** | **SD** | **Mean** | **SD** | **Mean** | **SD** | **Diet** | **Sex** | **Sex*diet** |
| **Body weight (kg)** | 46.4^aaa^ | 3.5 | 45.5^bbb^ | 11.4 | 12.1 | 1.3 | 12.4 | 0.9 | <0.001 | 0.88 | 0.57 |
| **Fat mass (kg)** | 19.9^aaa^ | 1.8 | 18.2^bbb^ | 6.3 | 2.0 | 0.2 | 1.9 | 0.4 | <0.001 | 0.29 | 0.64 |
| **Lean mass (kg)** | 25.8^aaa^ | 2.1 | 26.6^bbb^ | 5.1 | 9.8 | 1.2 | 10.1 | 0.6 | <0.001 | 0.46 | 0.51 |
| **BMD (g/cm2)** | 1.030^aaa^ | 0.082 | 0.999^bbb^ | 0.071 | 0.704 | 0.048 | 0.672 | 0.030 | <0.001 | 0.19 | 0.60 |
| **BMC (kg)** | 0.670^aaa^ | 0.092 | 0.636^bbb^ | 0.048 | 0.356 | 0.055 | 0.330 | 0.024 | <0.001 | 0.25 | 0.82 |
| **Body fat (%** | 42.8^aaa^ | 1.9 | 39.3^bbb^ | 3.9 | 16.6 | 1.8 | 15.4 | 2.2 | <0.001 | <0.05 | 0.27 |
| **Heart weight (g)** | 133.4^aaa^ | 17.0 | 131.9^bbb^ | 16.9 | 52.8 | 11.0 | 54.2 | 6.4 | <0.001 | 1.00 | 0.80 |
| **Relative heart weight (g/kg BW)** | 2.9^aaa^ | 0.3 | 3.0^bbb^ | 0.5 | 4.3 | 0.5 | 4.4 | 0.3 | <0.001 | 0.66 | 0.82 |
| **Kidney (g)** | 210.3^aaa^ | 20.7 | 192.8^bbb^ | 30.0 | 61.1 | 7.7 | 63.2 | 6.5 | <0.001 | 0.59 | 0.23 |
| **Relative kidney weight (g/kg BW)** | 4.5 | 0.4 | 4.4 | 1.0 | 5.1 | 0.5 | 5.1 | 0.6 | <0.05 | 0.86 | 0.68 |
| **Liver weight (g)** | 834.6^aaa^ | 146.8 | 805.6^bbb^ | 230.0 | 205.9 | 20.7 | 199.1 | 23.8 | <0.001 | 0.55 | 0.87 |
| **Relative liver weight (g/kg BW)** | 17.9 | 2.2 | 17.6 | 2.2 | 17.0 | 0.9 | 16.1 | 1.8 | 0.13 | 0.42 | 0.70 |

BMD: Bone mineral density, BMC: bone mineral content, BW: body weight, ns: not significant
